# CatBoost for big data: an interdisciplinary review

**DOI:** 10.1186/s40537-020-00369-8

**Published:** 2020-11-04

**Authors:** John T. Hancock, Taghi M. Khoshgoftaar

**Affiliations:** Florida Atlantic University, 777 Glades Road, Boca Raton, FL USA

**Keywords:** CatBoost, Big data, Categorical variable encoding, Ensemble methods, Machine learning, Decision tree

## Abstract

Gradient Boosted Decision Trees (GBDT’s) are a powerful tool for classification and regression tasks in Big Data. Researchers should be familiar with the strengths and weaknesses of current implementations of GBDT’s in order to use them effectively and make successful contributions. CatBoost is a member of the family of GBDT machine learning ensemble techniques. Since its debut in late 2018, researchers have successfully used CatBoost for machine learning studies involving Big Data. We take this opportunity to review recent research on CatBoost as it relates to Big Data, and learn best practices from studies that cast CatBoost in a positive light, as well as studies where CatBoost does not outshine other techniques, since we can learn lessons from both types of scenarios. Furthermore, as a Decision Tree based algorithm, CatBoost is well-suited to machine learning tasks involving categorical, heterogeneous data. Recent work across multiple disciplines illustrates CatBoost’s effectiveness and shortcomings in classification and regression tasks. Another important issue we expose in literature on CatBoost is its sensitivity to hyper-parameters and the importance of hyper-parameter tuning. One contribution we make is to take an interdisciplinary approach to cover studies related to CatBoost in a single work. This provides researchers an in-depth understanding to help clarify proper application of CatBoost in solving problems. To the best of our knowledge, this is the first survey that studies all works related to CatBoost in a single publication.

## Introduction

Modeling a system with regression or classification are common ways to scientifically investigate phenomena. Since Supervised Machine Learning ($\text {ML}$) [1] provides a way to automatically create regression and classification models from labeled datasets, researchers use Supervised $\text {ML}$ to model all sorts of phenomena in various fields. Hence, it is vital to stay informed on supervised $\text {ML}$ techniques practitioners currently use to achieve success. This is the first study that takes an interdisciplinary approach to reveal the emerging body of literature that shows CatBoost is an effective tool for use in supervised $\text {ML}$ techniques.

CatBoost is an open source, Gradient Boosted Decision Tree (GBDT) implementation for Supervised $\text {ML}$ bringing two innovations: Ordered Target Statistics and Ordered Boosting. We cover these innovations in detail in "CatBoost Gradient Boosted Trees Implementation" section. In the seminal paper on CatBoost, “Catboost: unbiased boosting with categorical features” [2], Prokhorenkova et al. recommend using $\text {GBDT}$ algorithms with heterogeneous data. They write, “For many years, it [gradient boosting] has remained the primary method for learning problems with heterogeneous features, noisy data, and complex dependencies: web search, recommendation systems, weather forecasting, and many others...” Heterogeneous datasets contain features with different data types. Tables in relational databases are often heterogeneous. The opposite of heterogeneous data is homogeneous data. Homogeneous data is data that is all the same type. For example, a dataset of features composed of pixel color intensity values is homogeneous. Such data may be multidimensional, but the components of each dimension are all the same type of data. Some works we survey here give empirical evidence for Prokhorenkova et al. claim that $\text {GBDT}$ algorithms yield better performance than other $\text {ML}$ algorithms on tasks for heterogeneous data. Other works we review show that $\text {GBDT}$ algorithms tend not to do as well as $\text {ML}$ alternatives such as neural networks on tasks involving homogeneous data. However, research into applying neural networks to heterogenous data [3, 4], is an active area of research. Therefore, researchers should give consideration to the nature of the data they intend to use for $\text {ML}$ implementations. It may be a mistake to consider only $\text {GBDT}$ algorithms if the data is homogeneous, and it may also be a mistake to ignore $\text {GBDT}$ algorithms if the data is heterogeneous.

In the interdisciplinary segment, we provide examples of experiments that will guide the reader in avoiding these mistakes. However, we feel the concept is important enough to merit immediate coverage here. Matsusaka et al. in “Prediction model of aryl hydrocarbon receptor activation by a novel qsar approach, deepsnap–deep learning” published a study that compares the performance of Gradient Boosted $\text {ML}$ algorithms to deep learning algorithms [5]. In their study, the authors report on the results of applying these algorithms to digital image data, that is, homogeneous data. The authors document that a deep learning algorithm gives better performance in terms of Area Under the Receiver Operating Characteristic Curve ($\text {AUC}$) and accuracy. This is not surprising to us, since Matsusaka et al. are evaluating the performance of these algorithms on homogeneous data. Matsusaka et al. results serve as a reminder to researchers applying $\text {ML}$ algorithms to homogeneous data to consider that gradient boosted algorithms may not be the best choice. Below, we cover multiple studies that confirm the same idea: CatBoost is a good solution for problems involving heterogeneous data, but may not be the optimal learner for problems involving homogeneous data. To put it succinctly, we find CatBoost is best suited to heterogeneous data.

Apart from the degree of heterogeneity of one’s data, a researcher working with Big Data [6, pp. 12–13] must also consider the time complexity of $\text {ML}$ algorithms. When working with large datasets, small differences in the time required to execute high frequency operations can result in large differences in the total time required to conduct experiments. Three studies we cover in detail, Prokhorenkova et al. [2], Spadon et al. [7] and Anghel et al. [8], show mixed results on the training time consumption of CatBoost and XGBoost [9]. We believe this is due to differences in hyper-parameters that the authors use to configure the learning algorithms. We also cover scenarios that show where researchers may trade running time for accuracy by using CatBoost or an alternative. Overall, we find the mixed results for running time complexity of CatBoost versus other learners that we hypothesize is rooted in CatBoost’s sensitivity to hyper-parameter settings.

**Fig. 1:**
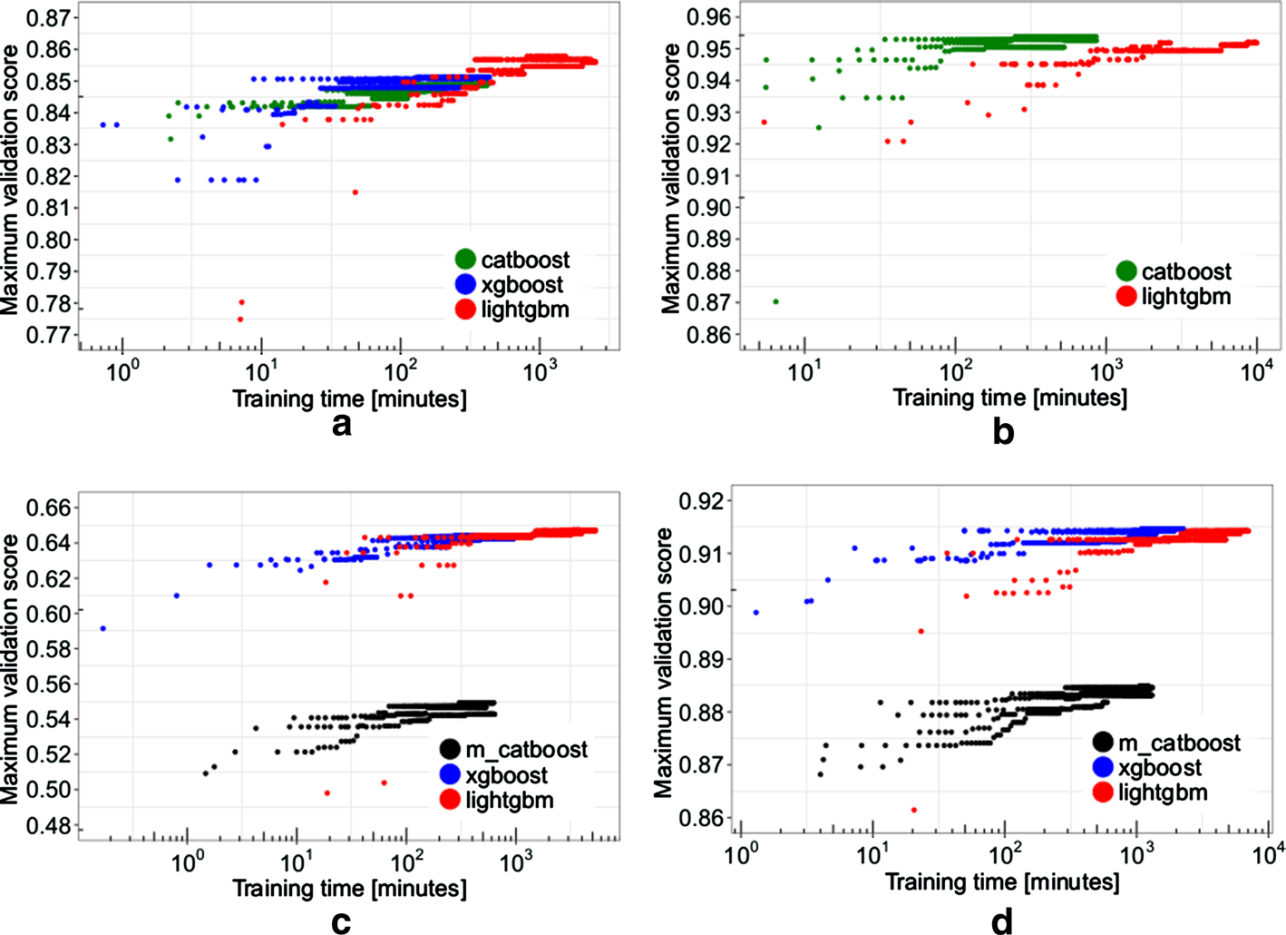
Image from [8] showing sensitivity of CatBoost to hyper-parameter settings; **a** records performance on the Higgs benchmark, **b** performance on the Epsilon benchmark, **c** performance on the Microsft benchmark, and **d** performance on the Yahoo Benchmark

**Fig. 2:**
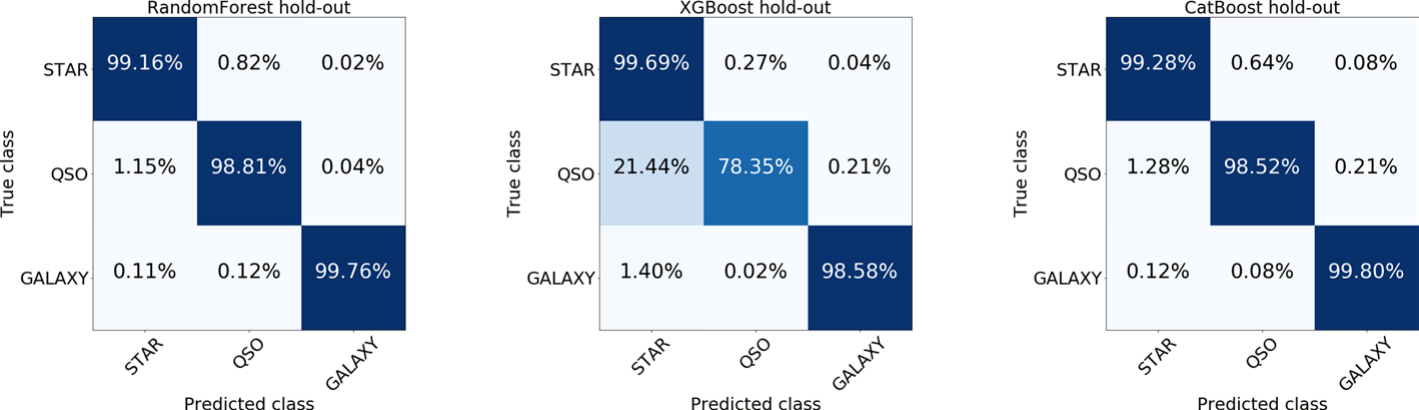
Confusion matrices from Khramtsov et al. showing the relative performance of Random Forest, CatBoost and XGBoost on the hold-out dataset [18]

We find one study that highlights CatBoost’s sensitivity to hyper-parameter settings that may shed some light on the discrepancies in the training time performance of CatBoost and other learners that we discover later in this review. This study is “Benchmarking and optimization of gradient boosting decision tree algorithms” by Anghel et al. [8]. In this study the authors document training time and accuracy for CatBoost, XGBoost, and LightGBM [10] on four benchmarking tasks involving large datasets. Figure 1, copied from [8, Fig. 2], contains plots of training times versus maximum validation scores during hyper-parameter optimization. It shows how training times vary widely as Anghel et al. change the algorithms’ hyper-parameters during optimization. We find Panel b interesting. This is where the authors report the results for the algorithms on the Epsilon^1^ benchmark. On the left side of Panel B, we see that some for some hyper-parameter configurations, CatBoost yields a maximum validation score sometime between 10 and 100 min, but for other configurations, it takes nearly 1000 min. In [2], Prokhorenkova et al. compare running time of CatBoost and XGBoost on a task involving the Epsilon dataset. However, XGBoost is missing from Panel b of Fig. 1. Anghel et al. report that they were unable to run XGBoost on the Epsilon benchmark due to memory constraints. That impediment to running XGBoost is an indication that, under the methods of their experiment, XGBoost consumes more memory than CatBoost for the same task. We include this result to emphasize that one may find it necessary to adjust CatBoost’s hyper-parameter settings in order to obtain the best results for CatBoost in terms of training time.

The application of GBDT algorithms for classification and regression tasks to many types of Big Data is well studied [11–13]. To the best of our knowledge, this is the first survey specifically dedicated to the CatBoost implementation of $\text {GBDT}$’s. Since its debut at the December 2018 Advances in Neural Information Processing Systems ($\text {NIPS}$) conference [2], researchers have conducted many experiments involving CatBoost. A number of these studies either involve Big Data, or techniques that will scale to Big Data. Hence, it is time for a review of these studies from a Big Data perspective. Researchers that work in Big Data environments often do so with a particular distributed framework, such as Apache Spark [14]. Some of these frameworks include GBDT implementations. For example, Spark MLlib’s GradientBoostedTrees module, [15], is one such implementation. For examples of GBDT applications in Spark please see [16] and [11] . However, as long as the distributed framework supports a language that the Gradient Boosted Decision Tree implementation has an application programming interface $\text {API}$ available for, it is possible to use that implementation in the framework; thus, freeing the user to select from the most appealing GBDT implementation available. For researchers wishing to employ CatBoost with very large datasets, one viable approach is to fit a CatBoost model to a representative sample using the CatBoost Python API, then apply a CatBoost model to the larger dataset using a distributed framework such as Spark or Hadoop [17] with CatBoost’s Java API. We provide this one example to show applying CatBoost to large datasets with popular distributed frameworks is feasible. However, we recognize that there exists a multitude of distributed frameworks suitable for Big Data that, in turn, support a myriad of programming languages. So, there should be many more valid approaches to applying trained CatBoost models to Big Data.

Researchers in disparate domains find applications for CatBoost. We find works in the fields of Astronomy [18], Finance [19–22], Medicine [23–26], Biology [27, 28], Electrical Utilities Fraud [29–31], Meteorology [32, 33], Psychology [34, 35], Traffic Engineering [7, 36], Cyber-security [37], Bio-chemistry [5, 38], and Marketing [39]. Therefore, a good understanding of CatBoost may provide one the opportunity to participate in interdisciplinary research. Our third finding is that the wide range of subjects where CatBoost is applicable is evidence that it is a general-purpose algorithm that behooves researchers to understand. On the other hand, as the works we survey demonstrate, CatBoost works better in some situations than others. We take an interdisciplinary approach to study different subject areas where researchers use CatBoost. For each of the subject areas we list here, we provide a section that details how researchers use CatBoost in that specific domain.

Before we cover applications of CatBoost in various domains, we discuss our search method, we cover related works, and then provide an overview of the GBDT ensemble technique, and the CatBoost implementation of GBDT’s. We touch on another GBDT implementation, LightGBM [10]. Like CatBoost, LightGBM has built-in support for encoding categorical variables. XGBoost is another GBDT implementation without built-in support for categorical features, so we choose not to give details on it. First of all, we provide details on the method we use to discover articles we cover.

### Search method

We used our University library database, Google Scholar [40], and the Web of Science [41] databases to search for the term “CatBoost.” We obtained results with 278 articles from OneSearch, 25 articles from Web of Science, and the first 100 results from Google Scholar. We then conducted a manual review of the 403 records resulting from the search. During the manual review we retained only the studies related to CatBoost and its applications. We do not include works where the authors mention CatBoost, but do not employ it in any experiment. We do not limit our search results to any specific subject area.

### Related work

In order to find related work, we review all studies retrieved using the search method detailed in the previous section, looking specifically for surveys on CatBoost. We did not find such a study. To the best of our knowledge, this is the first review that focuses exclusively on research involving the CatBoost implementation of GBDT’s. We therefore expanded our search for surveys on Gradient Boosted techniques, and find two related studies.

Prior to the introduction of CatBoost, Sagi and Rokach published “Ensemble learning: a survey” [42]. This work is broader in scope and covers ensemble methods in general. It was published in 2018, and includes a discussion of Gradient Boosted Decision Tree algorithms, but not CatBoost.

Another related work is “A survey of classification techniques in data mining” by Sujatha and Prabhakar [43]. This study also covers a broader range of $\text {ML}$ algorithms than what we cover here. Sujatha and Prabhakar published this study in 2013, prior to the release of CatBoost. Furthermore, it does not provide the depth of detail on GBDT algorithms that we go into here.

The absence of a survey of research where CatBoost is used, and the abundance of recent work involving CatBoost, indicates to us that a survey of these works is timely. A thorough understanding of GBDT’s and CatBoost is necessary before one delves into the different ways researchers apply CatBoost in various fields. Therefore, we continue with a review of GBDT’s and the CatBoost implementation of GBDT’s. After that, we conduct the interdisciplinary review, grouping coverage of works by field. From this perspective, one may see how to apply CatBoost given a problem in the same domain.

## Gradient Boosted Decision Trees

Jerome H. Friedman describes Gradient Boosting in the study titled “Greedy function approximation: a gradient boosting machine” [44]. In his paper, Friedman describes the Gradient Boosting $\text {ML}$ technique. Since it is a supervised $\text {ML}$ technique, we begin with a set $\left\{ {\mathbf {x}}_i, y_i\right\}$ of input values ${\mathbf {x}}_i$, and expected output values ${y_i}$, $i \in \left\{ 1 \ldots n\right\}$. Gradient boosting takes the approach of iteratively constructing a collection of functions $F^0, F^1, \ldots , F^t, \ldots , F^m$, given a loss function ${\mathcal {L}}\left( y_i, F^t\right)$. Here we would like to emphasize that ${\mathcal {L}}$ has two input values, the *i*th expected output value $y_i$, and the *t*th function $F^t$ that estimates $y_i$. Assuming we have constructed function $F^t$ we can improve our estimates of $y_i$ by finding another function $F^{t+1} = F^t + h^{t+1}\left( {\mathbf {x}}\right)$ such that $h^{t+1}$ minimizes the expected value of the loss function. That is,

1 $$\begin{aligned} h^{t+1} = \underset{h \in H}{\mathrm {argmin}} {\mathbb {E}} {\mathcal {L}}\left( y, F^t\right) . \end{aligned}$$

Where *H* is the set of candidate Decision Trees we are evaluating to choose one to add to the ensemble. Furthermore, by the definition of $F^{t+1}$, we can write the expected value of the loss function ${\mathcal {L}}$ in terms of $F^t$ and $h^{t+1}$:

2 $$\begin{aligned} {\mathbb {E}} {\mathcal {L}}\left( y, F^{t+1}\right) = {\mathbb {E}} {\mathcal {L}}\left( y, F^{t}+ h^{t+1} \right) \end{aligned}$$

One may notice that the right-hand side of Eq. (2) implies we wish to minimize the loss function’s value on *y* and $F^{t}$ plus *something*. If we assume ${\mathcal {L}}$ is continuous, and differentiable, we can add something related to the rate of change of ${\mathcal {L}}$ to $F^t$ to shift its value somewhere in the direction that ${\mathcal {L}}$ is decreasing. Therefore, if we set $h^{t+1}$ to values in the direction that the gradient of ${\mathcal {L}}$ with respect to $F^t$ is decreasing the fastest, we would have the $h^{t+1}$ that approximately minimizes ${\mathbb {E}} {\mathcal {L}}\left( y, F^t+h^{t+1}\right)$. Under these assumptions then we can write a reasonable approximation for $h^{t+1}$,

3 $$\begin{aligned} h^{t+1} \approx \underset{h \in H}{\mathrm {argmin}} {\mathbb {E}}\left( \frac{\partial {\mathcal {L}} y}{\partial F^t} - h \right) ^2. \end{aligned}$$

We refer to this technique as Gradient Boosting because we use the partial derivatives (gradients) of the loss function ${\mathcal {L}}$ with respect to the function $F^t$ to find $h^{t+1}$. Prokhorenkova et al. [2] point out that we may not have an easy way to compute $\underset{h \in H}{\mathrm {argmin}} {\mathbb {E}}\left( \frac{\partial {\mathcal {L}} y}{\partial F^t} - h \right) ^2$. This could be because it would be difficult, in general, to say what the probability of specific values of $\underset{h \in H}{\mathrm {argmin}} {\mathbb {E}}\left( \frac{\partial {\mathcal {L}} y}{\partial F^t} - h \right) ^2$ should be, and we may not know what $F^t$ should be because we could be using stochastic techniques, such as some algorithm to construct a Decision Tree to define $F^t$. However, we can assume, as Prokhorenkova et al. suggest,

4 $$\begin{aligned} \underset{h \in H}{\mathrm {argmin}} {\mathbb {E}}\left( \frac{\partial {\mathcal {L}} y}{\partial F^t} - h \right) ^2 \approx \underset{h \in H}{\mathrm {argmin}} \frac{1}{n}\left( \frac{\partial {\mathcal {L}} y}{\partial F^t} - h \right) ^2. \end{aligned}$$

Although we are covering Friedman’s Gradient Boosting Decision Trees technique in this section, we use this reference to Prokhorenkova et al. in our explanation, since our ultimate goal is to provide the reader a clear understanding of CatBoost.

We can take approximations (3) and (4) to obtain a concrete estimate for $h^{t+1}$:

5 $$\begin{aligned} h^{t+1} \approx \underset{h \in H}{\mathrm {argmin}} \frac{1}{n}\left( \frac{\partial {\mathcal {L}} y}{\partial F^t} - h \right) ^2. \end{aligned}$$

For GBDT’s the base case $F^0$ is a Decision Tree, and the $h^1, h^2, \ldots , h^t, \ldots h^m$ are also Decision Trees. When we add a Decision Tree to construct $F^{t+1}$ in this manner, the expected value of the loss function ${\mathbb {E}} {\mathcal {L}}\left( y, F^{t+1}\left( {\mathbf {x}}\right) \right)$ shrinks, implying that the estimates $F^{j+1}\left( {\mathbf {x}}_i\right)$ are better than the estimates $F^{j}\left( {\mathbf {x}}_i\right)$. CatBoost, as well as other currently popular GBDT techniques XGBoost and LightGBM, make refinements to the Gradient Boosting technique Friedman describes in [44]. Researchers who have a good understanding of how the GBDT technique works have a better chance of successfully applying it in any discipline. Similarly, researchers who know how CatBoost carries out the GBDT technique are better equipped to employ it in any domain. Therefore, we provide details on CatBoost in the next section.

### CatBoost Gradient Boosted Trees Implementation

In [2], Prokhorenkova et al. propose the CatBoost algorithm, and compare it with XGBoost and LightGBM. In their description of the CatBoost learner, they cover their refinements to the GBDT algorithm Friedman describes in [44]. Here we cover these refinements and some related hyper-parameters that users should be aware of since the related hyper-parameters’ values may also affect the resources CatBoost consumes.

CatBoost’s first refinement to Gradient Boosting is the manner in which it deals with high cardinality categorical variables. For low cardinality categorical variables, CatBoost uses one-hot encoding. The precise definition of low cardinality depends on the computing environment and whether the user is employing CatBoost in any specialized modes. The current version of CatBoost at the time of this writing, version 0.23.2, has a default value of 255 under some conditions when running on GPU’s, and 2 when running on CPU’s provided certain other specific conditions are not met. This is an obvious, yet non-trivial example of CatBoost’s sensitivity to hyper-parameters. One may obtain different results in terms of running time and other performance metrics since changing this hyper-parameter not only alters the type of processor CatBoost will use, but also the manner in which it will encode categorical features. We refer the reader to the CatBoost API documentation^2^ for further details on how CatBoost sets the threshold for one-hot encoding.

In [2], Prokhorenkova et al. use the term “Ordered Target Statistic” to refer to the technique CatBoost uses for encoding categorical variables, when CatBoost is not using one-hot encoding. Micci-Barreca introduces target statistics in “A preprocessing scheme for high-cardinality categorical attributes in classification and prediction problems” [45]. A target statistic is a value we calculate from the ground truth output values associated with particular values of a categorical attribute in a dataset. One strategy for dealing with categorical variables in $\text {ML}$ is to replace the categorical values of a feature with a target statistic.

The most important concept in the Ordered TS calculation is rooted in the distinction between training and test datasets. Let ${\mathcal {D}}$ be the set of all data available to train and evaluate our GBDT ensemble. The Decision Tree $h^{t+1}$ we add to the ensemble is the Decision Tree that minimizes the expected value of the loss function ${\mathbb {E}} {\mathcal {L}}$. We wish to use some data in ${\mathcal {D}}$ for fitting the Decision Tree $h^{t+1}$, and some data for finding the $h^{t+1}$ that minimizes ${\mathbb {E}} {\mathcal {L}}$. Our motivation for using the data in ${\mathcal {D}}$ in this way is to avoid what Prokhorenkova et al. define as “target leakage” [2]. We explain more on target leakage below; however, we finish our description of CatBoost’s encoding technique first. The way CatBoost chooses the data to use for fitting $h^{t+1}$ is to place an arbitrary order on the elements of ${\mathcal {D}}$ with a random permutation $\sigma$. Let $\sigma \left( k\right)$ be the *k*th element of ${\mathcal {D}}$ under $\sigma$, and ${\mathcal {D}}_k = \left\{ {\mathbf {x}}_1, {\mathbf {x}}_2, \ldots , {\mathbf {x}}_{k-1} \right\}$, ordered by the random permutation $\sigma$. CatBoost uses ${\mathcal {D}}_k$ as the data for fitting the Decision Tree $h^{t+1}$, and ${\mathcal {D}}$ as the data for evaluating whether $h^{t+1}$ is the Decision Tree that minimizes ${\mathbb {E}} {\mathcal {L}}\left( y, F^{t}+ h^{t+1} \right)$. The meaning of the notation ${\mathcal {D}}_k$ is the first important concept for understanding how CatBoost encodes the values of categorical variables.

The second important concept for understanding how CatBoost encodes the values of categorical variables is the indicator function $\mathbb {1}$. The indicator function $\mathbb {1}_{a=b}$ is a function of one variable *a* that has the value 1 when $a=b$, and 0 otherwise. The indicator function plays an important role in the formula CatBoost applies to map the values of a categorical feature to a numerical value. Specifically, this formula involves the indicator function $\mathbb {1}_{x_j^i=x_k^i}$. This indicator function takes the value 1 when the *i*th component of CatBoost’s input vector ${\mathbf {x}}_j$ is equal to the *i*th component of the input vector ${\mathbf {x}}_k$. Here we use *k* as in the *k*th element according to the order we put on ${\mathcal {D}}$ with the random permutation $\sigma$, and *i* takes on the integer values 1 through $k-1$.

Understanding these key concepts of the training data ${\mathcal {D}}$ and the indicator function $\mathbb {1}_{x_j^i=x_k^i}$, enables us to define the formula for the encoded value, ${\hat{x}}_i^k$, of the *i*th categorical variable of the *k*th element of ${\mathcal {D}}$ as:

6 $$\begin{aligned} {\hat{x}}_k^i = \frac{\sum _{x_j \in {\mathcal {D}}_k} \mathbb {1}_{x_j^i = x_k^i} \cdot y_j + a p}{\sum _{x_j \in {\mathcal {D}}_k} \mathbb {1}_{x_j^i = x_k^i} + a}. \end{aligned}$$

Prokhorenkova et al. define *p* as a prior commonly set to the average value of the label in the dataset, and *a* as a parameter greater than 0. We do not see a clear suggestion for the value of *a* [2]. However, one can see that setting *a* to a value greater than 0 in Eq. (6) ensures we will not divide by 0 in the case that none of the values $x_j^i$ equal $x_k^i$. Also, in that case, any value $a > 0$ guarantees ${\hat{x}}_k^i$ gets the value *p*.

CatBoost applies Eq. (6) when fitting the Decision Tree $h^{t+1}$, but uses a variation of it when evaluating $h^{t+1}$ to determine if it is *the* Decision Tree that minimizes ${\mathbb {E}} {\mathcal {L}}\left( y, F^{t}+ h^{t+1} \right)$. The variation on Eq. (6) is that instead of using the subset ${\mathcal {D}}_k$, it uses the entire set ${\mathcal {D}}$.

Now that we have an understanding of how CatBoost encodes categorical variables, we can understand why it uses this technique. As we mention above, CatBoost encodes categorical values in order to alleviate the problem of target leakage. Prokhorenkova *et al.* write that CatBoost avoids target leakage because the technique it uses for encoding categorical variables has a certain property, that they express in Eq. (7)

7 $$\begin{aligned} {\mathbb {E}} \left( {\hat{x}}^i |y=v \right) = {\mathbb {E}} \left( {\hat{x}}_k^i |y_k=v \right) . \end{aligned}$$

Interestingly, the way CatBoost’s encoding technique satisfies this property is to ensure we do *not* use the value $y_k$ in Eq. (6). Prokhorenkova et al. explain that if we use $y_k$ to encode features in ${\mathbf {x}}_k$ we create target leakage [2]. They define target leakage in terms of conditional shift. Noting that Eq. (7) involves conditional probabilities, we see that if Eq. (7) does not hold, it means that the expected value of all encoded values for the *i*th feature given a specific output value *v* does not equal the expected value of the encoded values for some training examples $\left( {\mathbf {x}}_k,y_k\right)$. In other words, when Eq. (7) does not hold, the expected encoded value ${\hat{x}}_k^i$ is shifted under the condition $y_k=v$. This is an overfitting condition in the sense that in the fitting process the model can exploit the correlation between ${\hat{x}}_k$ and $y_k$ during training, but the correlation will not exist during testing due to the difference in expected values when Eq. (7) does not hold. The way they suggest avoiding the shifting of the expected values under the conditions $y = v$ and $y_k = v$ is to exclude the value of $y_k$ in the computation of values for ${\hat{x}}^i$ when encoding the value $x_k^i$; hence, the definition of ${\mathcal {D}}_k$ above, and its role in computing the value of ${\hat{x}}_k^i$ in Eq. (6) above.

The second property of the Ordered TS that Prokhorenkova *et al.* describe is that it eventually uses all training examples $\left( {\mathbf {x}}_k, y_k\right)$. This property ensures that after sufficient iterations, we have encoded categorical values with all the information available in the training data. This second property balances the overfitting protection of the first property, to ensure we are not underfitting, because we are using all the available training data.

The way Prokhorenkova *et al*. enforce this property is another refinement to Gradient Boosting that they call the Ordered Boosting technique. Target leakage not only causes a conditional shift in the expected value of an encoded variable, but also it causes prediction shift in the expected value of the residuals we wish to minimize. To see why this is so, consider Approximation (5), and assume we are using CatBoost’s Ordered Target Statistic technique to encode some categorical variables to build the Gradient Boosted Decision Trees that constitute $F^{t+1}$. Then, because we are using Ordered Target Statistics to encode categorical variables, $\frac{\partial {\mathcal {L}} y}{\partial F^t}$ is also a random variable because we use the random permutation $\sigma \left( k\right)$ to choose the elements of $D_k$ to encode categorical variables that influence the value of $F^t$. Therefore, the distribution of $\frac{\partial {\mathcal {L}} y}{\partial F^t}$ can be shifted under the condition that we calculated $\frac{\partial {\mathcal {L}} y}{\partial F^t}$ with a particular encoding for $x_k^i$. Prokhorenkova *et al.* explain that this conditional shift leads to bias in the estimate we make for $h^{t+1}$, and that negatively impacts the metrics we obtain when evaluating of $F^{t+1}$ on data we did not use at training time. Prokhorenkova et al. refer to the impact on $F^{t+1}$ as its generalization ability. To combat this impact on $F^{t+1}$’s generalization ability, Prokhorenkova et al. propose Ordered Boosting. The key concept in Ordered Boosting is to use the same examples in ${\mathcal {D}}_k$ that we use to compute the Ordered Target Statistics, to compute the estimates for $h^{t+1}$, which means we must use them to compute the values of $\frac{\partial {\mathcal {L}} y}{\partial F^t}$. The reader will recall that ${\mathcal {D}}_k = \left\{ {\mathbf {x}}_1, {\mathbf {x}}_2, \ldots , {\mathbf {x}}_{k-1}\right\}$ depends on where we are at in iterating through the permutation $\sigma$ of the elements of ${\mathcal {D}}$. In other words, when we start with $k=1$, ${\mathcal {D}}_k$ will have one element in it. This means we will have a high variance in values we estimate for $\frac{\partial {\mathcal {L}} y}{\partial F^t}$. So, in Ordered Boosting, CatBoost uses multiple, independent permutations $\sigma _1, \sigma _2, \ldots , \sigma _s$ of ${\mathcal {D}}$ to compute a number of sets of residual values that it can use to find $h^{t+1}$, to obtain $F^{t+1}$, and maintain the guarantee that none of the values of $x_k^i$ are used to compute the values of the gradients $\frac{\partial {\mathcal {L}} y}{\partial F^t}$. At the same time using these multiple sets of residuals reduces the variance in CatBoost’s estimates of $\frac{\partial {\mathcal {L}} y}{\partial F^t}$. This is how Ordered Boosting avoids prediction shift.

Another important concept in CatBoost’s process of building Decision Trees is Oblivious Decision Trees ($\text {ODT}$’s). CatBoost constructs an ensemble of ODT’s. ODT’s are full binary trees, so if the ODT has *n* levels, it will have $2^n$ nodes. Furthermore, all non-leaf nodes of the ODT will have the same splitting criteria. To assist the reader’s understanding, in Table 1, we include a diagram of an ODT from Lou and Obukhov, “Bdt: gradient boosted decision tables for high accuracy and scoring efficiency” [46]. According to Prokhorenkova et al., ODT’s “...are balanced, less prone to overfitting, and allow speeding up execution at testing time significantly” [2]. We see ODT’s are balanced by definition. Since they are full binary trees the number of comparisons to reach a leaf node is the minimum number of comparisons to reach the maximum number of leaf nodes, so we agree that ODTs may yield more efficient executions than deeper Decision Trees that are not completely filled. The trade-off is that one must be careful in setting the maximum tree depth in CatBoost since the amount of memory CatBoost will use may grow by a factor of 2 times the number of trees in the ensemble for every unit of increase in the maximum tree depth. This is another example of CatBoost’s sensitivity to hyper-parameter settings that researchers should be aware of since it can have an impact on the amount of memory and running time their experiments consume. Perhaps the differences in running time complexity we see are rooted in improper values for this hyper-parameter.

**Table 1:**
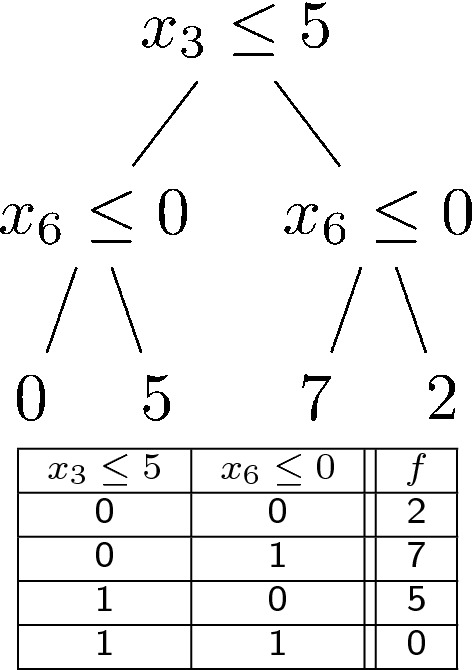
Oblivious Decision Tree example from Lou and Obukhov demonstrating a Decision Tree and Decision Table that provide equivalent logic [46]

Another useful feature CatBoost adds to GBDT’s is its support for interactions of features. Prokhorenkova et al. refer to these interactions as “feature combinations.” The authors claim CatBoost will greedily choose the most efficacious combinations of features during training [2]. Furthermore, CatBoost will use the Ordered TS method for encoding new features it generates from feature combinations when the combination of features includes a categorical variable. It is outside the scope of this survey to investigate whether other Decision Tree algorithms automatically discover new categorical features from combinations of other categorical features. To the best of our knowledge this is an innovation unique to CatBoost and another compelling reason for applying CatBoost in situations where one is working with data that has categorical features. Researchers should be aware of this functionality since it can impact the amount of time it takes for CatBoost to fit a GBDT model. One may control the maximum number of categorical features CatBoost will attempt to combine by setting a hyper-parameter value. This is another hyper-parameter value that CatBoost is sensitive to. Differences in the values researchers use for this hyper-parameter may account for some contradictory results we find in the literature on CatBoost.

CatBoost is an implementation of Gradient Boosted Decision Trees that avoids conditional shift with Ordered TS and prediction shift with Ordered Boosting. Both Ordered TS and Ordered Boosting rely on iterating through random permutations of a dataset. In Ordered TS we ensure that we do not use a specific training example $\left( {\mathbf {x}}_k, y_k\right)$ to encode any categorical component $x^i_k$ of ${\mathbf {x}}_k$. In Ordered Boosting, we use the same random permutation we use in Ordered TS to ensure we do not use the training example $\left( {\mathbf {x}}_k, y_k\right)$ to estimate the rate of change of the loss function we are using to improve (boost) the model’s estimate of $y_k$ for the input value ${\mathbf {x}}_k$. Also, in Ordered Boosting, we use several random permutations of our data simultaneously to reduce variance in our estimates of the rate of change of the loss function. CatBoost’s use of Ordered TS and Ordered Boosting make it a good choice for datasets with categorical variables that are sparse, or infrequently occur with specific target values, since these techniques guarantee that, given some unusual training example, CatBoost will involve other examples to update its estimate for the unusual example systematically. However, CatBoost is not the only GBDT algorithm that provides automatic encoding of categorical features. For a broad discussion on embedding techniques, please see [47]. In the next section we explain how a related GBDT implementation provides this functionality.

### LightGBM Support for Categorical Variables

LightGBM is another GBDT algorithm that supports automatic encoding categorical features. We discuss it here to give the reader an understanding of the difference in encoding techniques. Many of the works we include in this study compare the performance of LightGBM to CatBoost. The article that introduces LightGBM “Lightgbm: a highly efficient gradient boosting decision tree” by Ke *et al.*, does not mention support for categorical features [10]. However, LightGBM’s online documentation [48] states that LightGBM uses a technique from Fisher in the article “On grouping for maximum homogeneity” [49]. Interestingly, in the references in the study that introduces CatBoost [2], Prokhorenkova et al. also refer to the LightGBM on-line documentation, as well as the LightGBM source code when they mention LightGBM’s support for categorical features. Our point is that the LightGBM creators did not indicate they felt LightGBM’s handling of categorical variables was significant enough to document as a contribution in the article that introduces LightGBM. Fisher’s technique, that LightGBM uses, partitions a set of arbitrary numbers into subsets with minimum variance between members of the partitions. LightGBM applies Fisher’s technique when evaluating the splitting criteria for a categorical feature as it is growing a Decision Tree. LightGBM constructs a histogram of the values of a categorical feature, then sorts the histogram by gradient statistics. It then iterates through the sorted histogram to divide the set of values of the categorical feature into two groups. Prokhorenkova et al. criticize the technique LightGBM uses for two reasons. The first is that the technique requires more memory than using Ordered $\text {TS}$ because LightGBM must maintain gradient statistics for all possible values of a categorical feature. The second issue Prokhorenkova et al. point out is that it is computationally expensive for LightGBM to compute the gradient statistics necessary to build the sorted histogram. Furthermore, Prokhorenkova et al. also point out that in LightGBM’s documentation [50], the authors appear to back away from recommending LightGBM’s technique for handling categorical features. We understand this to mean that the LightGBM documentation’s authors caution the user not to use LightGBM support for high-cardinality categorical features.

With CatBoost and LightGBM both being GBDT implementations, and both providing similar functionality to support automatic handling of categorical features, one may be curious to see how these learners perform when compared against each other on the same $\text {ML}$ task. Moreover, one may wish to know the same for other types of learners as well. Many studies we include document experiments to answer this question. Hence, in the next section, we provide tables that summarize the outcomes of these experiments. Studies are grouped by field in these tables. Perhaps it is disappointing that there is no clear winner in all situations. However, this fact motivates us to investigate the details of these works to see why this is the case in the sections following the tables.

## CatBoost applications by field

### Tables of works studied

A good measure of the generality of an idea is its applicability in diverse settings. We mention existing surveys on applications of $\text {GBDT}$ algorithms in our section on related works. This shows that $\text {GBDT}$ algorithms are rooted in a general idea. We review studies where the authors use CatBoost in a wide array of fields. Hence, CatBoost is implemented in such a way that it preserves the generality of Friedman’s conception of the $\text {GBDT}$ algorithm. First, we summarize the works we cover in Tables 2, 3, 4, 5, 6, 7, 8, 9, 10, 11 and 12. Then, we cover them in detail, grouped by the fields the works contribute to.

**Table 2:** Machine learning

Title	CatBoost: unbiased boosting with categorical features
Description	Paper introducing CatBoost algorithm
Performance metric	logloss, zero-one loss
Winner	CatBoost
Reference	[2]
Title	Benchmarking and optimization of gradient boosting decision tree algorithms
Description	Compare CatBoost, LightGBM, and XGBoost run on GPU’s, using four benchmark tasks
Performance metric	AUC ROC and Normalized discounted cumulative gain ($\text {NDCG}$)
Winner	CatBoost wins AUC for Epsilon DataSet, LightGBM wins AUC for the Higgs dataset, XGBoost wins (NDCG) for Microsoft and Yahoo Datasets
Reference	[8]

**Table 3:** Traffic engineering

Title	A Semi-Supervised Tri-CatBoost method for driving style recognition
Description	Combine labeled and unlabeled data, use CatBoost as a base classifier to identify driving style
Performance metric	N/A CatBoost used for semi-supervised learning not compared to other classifiers
Winner	N/A
Reference	[36]
Title	Reconstructing commuters network using machine learning and urban indicators.
Description	Construct graph on human movement between cities, extract features, apply CatBoost among other algorithms to reconstruct graph
Performance metric	Accuracy
Winner	CatBoost wins but training time is long compared to XGBoost, so authors use XGBoost for remainder of study
Reference	[7]

**Table 4:** Finance

Title	Comparison between XGBoost, LightGBM and CatBoost using a home credit dataset
Description	Evaluate of XGBoost, LightGBM, and CatBoost performance in predicting loan default
Performance metric	AUC, running time
Winner	LightGBM
Reference	[19]
Title	Short term electricity spot price forecasting using CatBoost and bidirectional long short term memory neural network
Description	CatBoost for feature selection for time-series data
Performance metric	Mean absolute percentage error
Winner	CatBoost not a competitor, used for feature selection
Reference	[21]
Title	Research on personal credit scoring model based on multi-source data
Description	Use “Stacking&Blending” with CatBoost, Logistic Regression, and Random Forest to calculate credit score in a regression technique
Performance metric	Model is ensemble of no direct comparison between algorithms; performance measured in AUC
Winner	N/A
Reference	[22]
Title	Predicting loan default in peer-to-peer lending using narrative data.
Description	Evaluate CatBoost against other classifiers on the task of predicting loan default using Lending Club data
Performance metric	Accuracy, AUC, H measure, type I error rate, type II error rate
Winner	CatBoost
Reference	[20]

**Table 5:** Astronomy

Title	KiDS-SQuaD II. Machine learning selection of bright extragalactic objects to search for new gravitationally lensed quasars
Description	Use CatBoost to classify astronomical data
Performance metric	AUC
Winner	CatBoost
Reference	[18]

**Table 6:** Cyber-security

Title	Attack detection in enterprise networks by machine learning methods
Description	Compare CatBoost, LightGBM, SVM, and logistic regression in multi-class and binary classification task of identifying computer network attacks.
Performance metric	AUC, CV balanced accuracy, balanced accuracy, F1, precision, recall
Winner	CatBoost
Reference	[37]

**Table 7:** Meteorology

Title	Short-term weather forecast based on wavelet denoising and catboost
Description	Use CatBoost to predict weather-related observations, and compare to other machine learning algorithms doing the same task
Performance metric	unique method, based on root mean squared error
Winner	CatBoost
Reference	[51]
Title	Evaluation of CatBoost method for prediction of reference evapotranspiration in humid regions
Description	compare CatBoost, $\text {SVM}$, and $\text {RF}$ ability to predict amount of water lost through evaporation and transpiration
Performance metric	MAPE, RSME, R^2^
Winner	Results do not indicate clear overall-winner
Reference	[33]

**Table 8:** Medicine

Title	The use of data mining methods for the prediction of dementia: evidence from the english longitudinal study of aging
Description	Classify dementia on imbalanced data, maximum cardinality of feature is 50, compare CatBoost to other classifiers
Performance metric	Normalized Gini coefficient
Winner	Convolutional neural network
Reference	[26]
Title	A novel fracture prediction model using machine learning in a community-based cohort
Description	Use CatBoost to predict fragility fracture
Performance metric	AUC
Winner	CatBoost
Reference	[24]
	An efficient novel approach for iris recognition based on stylometric features and machine learning techniques
Description	Use CatBoost after doing feature extraction from image data converted to base-64 encoded data
Performance metric	AUC
Winner	multiboostAB
Reference	[23]

**Table 9:** Biology

Title	CT-based machine learning model to predict the Fuhrman nuclear grade of clear cell renal cell carcinoma
Description	Classify kidney cancer images into instances of high-grade or low-grade cancer, presents opportunities for research at Big Data scale
Performance metric	Used only CatBoost
Winner	N/A
Reference	[28]
Title	diseases spread prediction in tropical areas by machine learning methods ensembling and spatial analysis techniques
Description	Use CatBoost to predict spread of dengue fever
Performance metric	Mean absolute error
Winner	LSTM and XGBoost ensemble
Reference	[27]
Title	Performance analysis of boosting classifiers in recognizing activities of daily living
Description	Compare CatBoost with XGBoost in ability to identify human physical activity types from sensor data
Performance metric	f-measure
Winner	Friedman stochastic gradient boosting, ada-decision trees
Reference	[25]

**Table 10:** Marketing

Title	Predicting online shopping behavior from clickstream data using deep learning
Description	CatBoost is part of ensemble that is best clickstream predictor
Performance metric	AUC
Winner	GRU—CatBoost Ensemble
Reference	[39]

**Table 11:** Bio-chemistry

Title	Construction and analysis of molecular association network by combining behavior representation and node attributes.
Description	Leverage graph representation of association network of biological entities to predict associations as input for classifier, compare CatBoost with other popular classifiers as association predictor
Performance metric	Accuracy, sensitivity, specificity, precision, Matthew’s Correlation, Coefficient, AUC,
Winner	CatBoost (except Sensitivity)
Reference	[38]
Title	Prediction model of aryl hydrocarbon receptor activation by a novel QSAR approach, deepSnap–deep learning
Description	Compare CatBoost to other learners in image processing task related to study relationship between genes and liver function
Performance metric	AUC, accuracy
Winner	DeepSnap-DL (deep learning algorithm)
Reference	[5]

**Table 12:** Electrical utilities fraud

Title	Bridging the gap between energy consumption and distribution through non-technical loss detection
Description	Use CatBoost for predicting non-technical loss in power distribution networks, authors report little in terms of quantitative results
Performance metric	Performance metric not explicit
Winner	Not clear, authors do not give exact numbers
Reference	[29]
Title	Performance Analysis of Different Types of Machine Learning Classifiers for Non-Technical Loss Detection
Description	Compare CatBoost with 14 other classifiers
Performance metric	Precision, recall, F-Measure
Winner	CatBoost has highest precision and F-measure, $\text {ANN}$ has 0.003 higher recall
Reference	[52]
Title	Energy theft detection using gradient boosting theft detector with feature engineering-based preprocessing
Description	Technique for using CatBoost with highly imbalanced data
Performance metric	True positive rate, false positive rate
Winner	CatBoost, has lowest false positive rate, LightGBM wins true positive rate, CatBoost has longest total train and test time, LightGBM has shortest total train and test time
Reference	[31]
Title	Impact of feature selection on non-technical loss detection
Description	Use incremental feature selection, compare performance of CatBoost, Decision Tree and K-Nearest Neighbors classifiers
Performance metric	Precision, recall, F-Measure
Winner	CatBoost, except for recall of models trained with 9 features, where K-NN wins
Reference	[30]

In the sections that follow, we organize studies involving CatBoost by subject. As we review works in specific fields, general techniques that apply in multiple disciplines become apparent. We take an objective look at CatBoost’s performance in many applications to show where it is a good choice, and where it is not. The first subject area we cover is the field of Astronomy.

### Astronomy

Published in 2012 [53], The Kilo Degree Survey $\text {KiDS}$, de Jong et al. is the result of an Astronomical study that researchers for the European Southern Observatory ($\text {ESO}$) carried out using equipment in the Very Large Telescope installation in Chile. According to de Jong et al. the full KiDS survey is 15 terabytes ($\text {TB}$) of data. Given such a volume of data, some form of automation is necessary to conduct research on it. Supervised $\text {ML}$ is one way to approach data of this magnitude. One such study that takes the Supervised $\text {ML}$ approach specifically with CatBoost is “Kids-squad ii. machine learning selection of bright extragalactic objects to search for new gravitationally lensed quasars”, by Khramtsov et al. [18]. In this article, the authors use CatBoost to classify objects in $\text {KiDS}$ data into the categories: stars, quasi-stellar radio sources ($\text {quasar}$), and galaxies. After they classify objects in $\text {KiDS}$ data, Khramtsov et al. take on the task of creating a catalog of gravitationally lensed quasars. Gravitational lensing refers to the alteration of an object’s appearance when the light from it is bent as it travels through the gravitational field of another massive object. According to Khramtsov et al. gravitationally lensed quasars are interesting for astronomers because they can be used for studying the expansion history of the universe, dark matter around galaxies, and planets outside our solar system.

In order to create a training sample, Khramtsov et al. use data from an earlier study, the Sloan Digital Sky Survey ($\text {SDSS}$) [54], as a source for labels. They create a training dataset from the data on objects that are present in both $\text {SDSS}$ and $\text {KiDS}$. Their training dataset has 127,376 instances.

The features the authors use are derived from color intensity measurements of objects in the KiDS data. The data contain nine optical or infrared colors, and Khramtsov et al. mention that they also use 36 combinations of pairs of colors, and one continuous feature CLASS_STAR, that takes a value from 0 to 1, that gauges how point-like a light source is. Khramtsov et al. write that they perform classification in a 37 dimensional features space, which implies they discard the 9 original color intensity features after they derive the 36 features from pairs of color intensity features. The total number of instances in the $\text {KiDS}$ data that they classify is approximately 9.6 million. Khramtsov et al. report that their $\text {KiDS}$ data is imbalanced, since their final classification finds 5,665,586 galaxies, 3,660,368 stars, and 145,653 quasars, and 122,306 instances of indeterminate class. For in-depth coverage of techniques for addressing class imbalance, please see [55]. Khramtsov et al. apply threshold values to the list of output probabilities of the CatBoost classifier to partition $\text {KiDS}$ data into classes. The indeterminate instances are those where the probability that the instance is a quasar or a galaxy are approximately the same.

Khramtsov et al. evaluate three different Decision Tree based ensemble techniques for classifying their labeled data: CatBoost, XGBoost, and Random Forest. Please see Fig. 2 for confusion matrices of each classifier’s performance on the hold-out dataset. We find the confusion matrices for Random Forest and CatBoost to be quite similar. However, Khramtsov et al. find CatBoost yielded the best performance for the classification task. In our discussion on CatBoost, we write that Prokhorenkova et al. claim that Oblivious Decision Trees are less prone to overfitting. Khramtsov et al. provide some empirical evidence for the claim in Appendix A.3, where they report that CatBoost, “...is more able to generalize good results on an unseen dataset.” [18] Their justification for this statement is that on the hold-out dataset, CatBoost yields a smaller difference in Matthews’ Correlation Coefficient ($\text {MCC}$) between training and hold-out datasets.

In one study on Astronomy Khramtsov et al. make a clear case for using CatBoost. For their purposes, CatBoost is the best classifier for detecting quasars in the $\text {KiDS}$ data. Khramtsov et al. follow a pattern we see in many of the fields we cover; they compare performance of multiple learners to select the best. In the studies that we cover next we see that CatBoost is usually a strong contender among GBDT implementations, and sometimes with other unrelated classification or regression $\text {ML}$ algorithms. Overall, Khramtsov et al. study is a good example of how CatBoost works well with heterogeneous, categorical Big Data. At first glance, one may be tempted to think KiDS data is homogeneous since it consists of color intensities, but the CLASS_STAR feature makes the data heterogeneous. In the next section, we focus on the subject of applications of CatBoost to Finance.

### Finance

We find studies such as “Comparison between xgboost, lightgbm and catboost using a home credit dataset” by Essam Al Daoud that compare the performance of CatBoost to other algorithms in Finance-related $\text {ML}$ tasks [19]. This study compares XGBoost, LightGBM, and CatBoost on the $\text {ML}$ task of predicting loan repayment given a list of features describing the borrower. Daoud reports that LightGBM yields the best performance in terms of AUC and training time. We do not see where Daoud provides a reference for the source of the Home Credit Dataset. Moreover, Daoud does not list hyper-parameters used in the experiments in [19], so Daoud’s results may be difficult to reproduce. For example, one important hyper-parameter for CatBoost is iterations, that specifies the maximum number of Decision Trees CatBoost will construct. A low value for this parameter can impact CatBoost’s performance relative to other GBDT implementations. Since Daoud does not document this value, it is difficult to conclude that LightGBM is the best performer. We recommend as a best practice that researchers document hyper-parameters used in GBDT implementations they compare.

Daoud compares CatBoost to other GBDT implementations, but researchers have also explored blending CatBoost with other models. “Research on personal credit scoring model based on multi-source data” by Zhang et al. is a study where the authors classify borrowers into groups of those with overdue payments on their loans, and those who pay on time [22]. Zhang et al. do not name the dataset they use, but it appears to be different from the dataset Daoud uses since it has a different number of instances and is data from Chinese credit markets. Zhang et al. employ CatBoost, Random Forest, and Logistic Regression together using the “Stacking&Blending” method. The Stacking&Blending method is a linear combination of the outputs of CatBoost, Random Forest, and Logistic Regression. Zhang et al. report that with the Stacking&Blending technique, they determine the weights for the linear combination according to the accuracy of individual models, but they do not give any detail on precisely how the weights are calculated. It could be the case that the authors selected weights manually according to what gave the best result for their study. They write, “The $w_{ij}$ used in this paper is determined by the accuracy of multiple trainings of a single model. The higher the accuracy of a single model, the greater the weight of the model.” $w_{ij}$ is constant for any value of *j*, the index of the sample, but changes for *i* which is the index of the model. The weights they list are: 0.2 for Logistic Regression, 0.4 for random forest, and 0.4 for CatBoost. If Zhang et al. did not use a computational approach to find the values for these weights, there is an opportunity for further research to use an optimization technique to show what the best values for coefficients would be to use. Zhang et al. do not compare the performance of their blended model to any other model. Nevertheless, this work shows CatBoost is an effective component of a system for predicting when a borrower will have overdue payments. Their technique has an overall AUC of 0.73. While they show their Stacking&Blending technique has an AUC that is far better than what a random guess would yield, we do not see a direct comparison of this technique to other learners in their study. Zhang et al.’s study is a demonstration of the Stacking&Blending technique; however, the next finance-related study we cover is a more comprehensive comparison of more widely used techniques we find to be more informative.

We return to the task of predicting outright loan default with “Predicting loan default in peer-to-peer lending using narrative data”, by Xia et al. [20]. This study is an evaluation similar to Daoud [19] that showcases CatBooost’s ability to predict when a borrower will default on a loan. Xia et al. work with data from Lending Club^3^, an online platform that connects individuals to facilitate making personal loans.

In their experiments, Xia et al. extract what they call “hard information” and “soft information” from data that Lending Club makes available to the public. In the context of their study, hard information is numerical data from loan applications: loan parameters, the applicant’s creditworthiness, and the applicant’s solvency. The soft information that Xia et al. work with relates to free-form text on the loan application. This includes: the number of times someone edits the loan description, number of words in the loan description, and a one-hot encoded feature calculated from the words in the loan description. The one-hot encoded feature is calculated from the output of a clustering algorithm, that is composed with the Skip-Gram variant of Word2Vec [56], which is in turn composed with term frequency-inverse document frequency ($\text {TF-IDF}$) applied to the loan description text [57]. Xia et al. derive three datasets of hard and soft information from the Lending Club data, one dataset for each of the years 2011, 2012, and 2013.

The performance of different algorithms is compared to that of CatBoost. The algorithms used for comparison are: Logistic Regression ($\text {LR}$), Regression Tree ($\text {RT}$), Bagging Neural Network ($\text {BNN}$), Random Forest ($\text {RF}$), $\text {GBDT}$’s, and XGBoost. All of these algorithms are available in the Python Scikit-learn library [58] except for XGBoost. Their results show that combining their feature extraction technique with CatBoost yields the best performance in terms of accuracy, ($\text {AUC}$), H-measure [59] type I error rate, and type II error rate. It is also interesting to note that Xia et al. report using a Bayesian hyper-parameter tuning method. This could be a factor in why Xia et al. find CatBoost yields the best performance whereas Daoud does not in similar machine learning tasks.

We reproduce a part of [20, Tab. 7] in Table 13 below that shows the superior performance of CatBoost for predicting loan defaults in 2013 Lending Club data. Xia et al. also report the results of significance tests that show CatBoost’s superior performance is statistically significant. Metrics related to the running times of training or testing the models they compare are not reported in their study. They also do not report the running time performance of their feature extraction technique. Therefore, we see an opportunity for future research in evaluating these running times. This research is relevant in the field of Big Data since, with large datasets, one might be willing to trade off running time for performance metrics. Xia et al. results indicate CatBoost can be a good choice for predicting when a candidate borrower will default on a loan.

**Table 13:** From [20], bracketed numbers are confidence intervals; *note* we do not find where Xia et al. document the significance level for the confidence intervals; here “softer” means models are trained with all available features

Softer dataset			
Model	Accuracy	AUC	H-measure
LR-softer	0.7516 [0.7508, 0.7523]	0.6151 [0.6139, 0.6163]	0.0843 [0.0827, 0.0860]
RT-softer	0.6952 [0.6911, 0.6996]	0.5444 [0.5391, 0.5493]	0.0124 [0.0095, 0.0153]
BNN-softer	0.7496 [0.7480, 0.7516]	0.6120 [0.6095, 0.6151]	0.0801 [0.0766, 0.0843]
RF-softer	0.7436 [0.7415, 0.7456]	0.6043 [0.6013, 0.6073]	0.0695 [0.0659, 0.0733]
GBDT-softer	0.7504 [0.7488, 0.7520]	0.6132 [0.6107, 0.6158]	0.0818 [0.0784.0.0853]
XGBoost-softer	0.7511 [0.7496, 0.7526]	0.6143 [0.6120, 0.6167]	0.0833 [0.0801, 0.0866]
CatBoost-softer	0.7523 [0.7511, 0.7535]	0.6162 [0.6144, 0.6180]	0.0859 [0.0834, 0.0885]

Model	Type I rate	Type II rate	
LR-softer	0.1557 [0.1550, 0.1565]	0.6142 [0.6123, 0.6160]	
RT-softer	0.2024 [0.1978, 0.2072]	0.7087 [0.6994, 0.7198]	
BNN-softer	0.1569 [0.1557, 0.1580]	0.6190 [0.6141, 0.6231]	
RF-softer	0.1617 [0.1599, 0.1639]	0.6298 [0.6241, 0.6346]	
GBDT-softer	0.1564 [0.1554, 0.1574]	0.6171 [0.6130, 0.6211]	
XGBoost-softer	0.1560 [0.1550, 0.1569]	0.6153 [0.6115, 0.6190]	
CatBoost-softer	0.1552 [0.1545, 0.1560]	0.6124 [0.6095, 0.6152]	

Moving on from the subject of using CatBoost to classify borrowers, another finance-related study involving CatBoost is “Short term electricity spot price forecasting using catboost and bidirectional long short term memory neural network” by Zhang and Fleyeh [21]. The role of CatBoost in this study is that of a feature selector. Zhang and Fleyeh use historical electricity futures prices time-series data as well as the categorical values: day of week, hour of day, and a weekend/ not weekend indicator. Zhang and Fleyeh also do autocorrelation analysis of time series data to discover which previous price data are likely predictors. They then use feature importance scoring functionality, which is part of the CatBoost software package, to determine which features to extract from their raw data to use as input to other $\text {ML}$ algorithms. Using CatBoost as a feature selector to rank features is an interesting approach for researchers working with Big Data, since some datasets that qualify as Big Data have large numbers of features. CatBoost provides a way to automatically select features. Zhang and Fleyeh propose a novel composition of CatBoost and Bidirectional Long Short Term Memory ($\text {BDLSTM}$) [60] but they do not compare CatBoost as a feature selector to any other GBDT, or any other feature selection technique. Therefore, there is an opportunity for future research to compare the efficacy of different feature selection techniques in Zhang and Fleyeh’s technique for forecasting electricity spot prices. CatBoost’s built-in support for encoding categorical features makes it a convenient choice for a feature selection technique.

For finance-related studies involving CatBoost, we find research that is mostly credit-related. Zhang and Fleyeh’s use of CatBoost for feature selection is the exception to that rule. Therefore, there are opportunities for researchers to apply CatBoost to other sub-domains in finance, and be the first to do so. That CatBoost and LightGBM do well on the task of loan default prediction supports Prokhorenkova’s claim that GBDT’s do well for problems involving heterogeneous data. Also, Xia et al. use of a hyper-parameter optimization technique may explain why CatBoost outperforms LightGBM in Xia et al. experiments, but not in Daoud’s experiments. The next field we delve into is Medicine, and the first work we study in that field has something in common with [22]—it describes another blending technique.

### Medicine

“The use of data mining methods for the prediction of dementia: evidence from the english longitudinal study of aging” by Yang and Bath is a study that evaluates various combinations of classifiers [26]. The classifiers they compare are CatBoost, XGBoost, Logistic Regression, Regularized Greedy Forests ($\text {RGF}$) [61], and Convolutional Neural Networks ($\text {CNN}$) on the task of classifying individuals as having dementia, or not having dementia according to data available on them from the English Longitudinal Study on Aging $\text {ELSA}$ [62] dataset. The models are combined with an ensemble technique, similar to one Zhang et al. use in [22]. The final output of the ensemble is

8 $$\begin{aligned} y=\sum _{i=1}^n w_ix_i \end{aligned}$$

where $x_i$ is the output of the *i*th model in the ensemble, and the $w_i$ coefficients are subject to the constraint that they must sum to 1. Also, like Zhang *et al.* in [22], Yang and Bath do not provide details on how the $w_i$ are calculated. This provides further motivation for research that elucidates how one might go about optimizing weights for ensembles involving CatBoost; since we have examples of research missing this information in two disparate fields – Finance, and here, Medicine. The evaluation metric the authors use is the Normalized Gini Coefficient, defined as $2 \times AUC -1$. The Normalized Gini Coefficient has a scale from 0 to 1, but is nothing more than a linear transformation of $\text {AUC}$. The algorithms used for addressing class imbalance in the data are Synthetic Minority Over-sampling Technique ($\text {SMOTE}$) [63] and Adaptive Synthetic Sampling Approach ($\text {ADASYN}$) [64]. In addition, the authors use hyper-parameters for the various classifier implementations to address class imbalance. We feel using these parameters in addition to SMOTE or ADASYN would be redundant, since these algorithms balance the data before it is presented to a classifier. For further studies on application of techniques for addressing classs imbalance see [65] and [6].

Yang and Beth report that the data they use in their study is imbalanced with 142 out of 9666 records in the positive class for one dataset they use, and 109 out of 8445 records in the positive class for another dataset. This is clearly not a study in Big Data since the dataset is small in comparison to others we cover in this study. However, we choose to cover the study here because Yang and Bath’s ensemble technique is interesting and applicable to larger datasets.

Yang and Bath perform a set of experiments, where the number of features and the combination of classifiers are factors in the experiments. In the study, the authors report that the raw data has 400 features. The authors include a supplementary spreadsheet that describes the data they use, and we find a maximum of 50 for the number of levels a feature can obtain. Furthermore, we can see that many of the features are categorical and heterogeneous with descriptions such as “father has diabetes” or “difficulty taking medications.” As we mentioned earlier, the default value for the minimum number of features CatBoost may use for encoding categorical variables with Ordered $\text {TS}$ may be 255 if one runs CatBoost on GPU’s, so it could be the case that Yang and Bath did not take advantage of Ordered $\text {TS}$ encoding of categorical variables, which could have had an impact on their results. Yang and Bath report on the performance of each learner separately, and several combinations in different ensembles. We include copies of both results in Tables 14 and 15. The tables show that CatBoost is not the best performing learner separately, and it is also not a member of the best performing ensemble. However, we do not find a significance test that shows the results of different classifiers is statistically significant. We choose to include this study since it highlights the use of CatBoost in the field of medicine, and techniques used are applicable in Big Data problems. The next item in our study is also in the medical field, but focuses on predicting a different sort of condition.

**Table 14:** “Best Gini scores of individual ML algorithms on the test data” [26]

XGB	LGB	CatBoost	K-CNN	RF	RGF	LR
0.9234	0.9153	0.9218	0.9307	0.9295	0.9276	0.9069

XGB stands for XGBoost; LGB for LightGBM; K-CNN the Keras [66] implementation of Convolutional Neural Networks; RF for Random Forest; RGF for Regularized Greedy Forest; performance in terms of Normalized Gini Coefficient

**Table 15:** From Yang and Bath [26], “System performance on the test data using different ensemble strategies”

E1	E2	E3	E4	E5	E6
0.9332	0.9331	0.9325	0.9322	0.9332	0.9333

E1 is ensemble of K-CNN, RF and RGF; E2 is ensemble of K-CNN, RF and XGB; E3 is ensemble of K-CNN, RGF, XGB; E4 is ensemble of K-CNN, RGF and CatBoost; E5 is ensemble of K-CNN, RF, RGF and CatBoost; E6 is ensemble of K-CNN, RF, RGF and XGB; performance in terms of Normalized Gini Coefficient

“A novel fracture prediction model using machine learning in a community-based cohort”, by Kong et al. is a study on using ML on the task of predicting fragility fractures in patients [24]. Fragility fractures are bone fractures that occur from little or no trauma. The authors report that their study uses a cohort of 2227 patients. We assume there is one sample in the dataset per participant, so this is not a study in Big Data. However, we choose to include their study in our survey because it shows how CatBoost can outperform other ML algorithms in tasks involving heterogeneous, categorical data. Assuming the list of clinical characteristics of participants listed in [24] [Tab. 1] is the comprehensive list of features, there are 35 features in the dataset, 9 of which we consider to be categorical. However, the authors also report results for models using the top 20 most important features. The authors determine feature importance using Shapley additive explanations ($\text {SHAP}$) analysis [67]. Results show CatBoost outperforms Support Vector Machine ($\text {SVM}$) and Logistic Regression in the task of identifying participants who will develop fragility fractures. CatBoost outperforms $\text {SVM}$ and Logistic Regression in all experiments. We focus on the key result of predicting all types of fragility fractures, using all features in the cohort data. For this result CatBoost yields an AUC of 0.688, whereas Logistic Regression yields an AUC of 0.614, and $\text {SVM}$ yields an AUC of 0.500. The $\text {AUC}$ that $\text {SVM}$ yields is no better than a model that randomly classifies inputs into categories. This is interesting because in the next study we cover [23], the authors use data with features that are derived from heterogeneous numerical data. In that study two algorithms related to $\text {SVM}$, Sequential Minimal Optimization ($\text {SMO}$) (which is the name for the $\text {SVM}$ implementation in the Weka [68] $\text {ML}$ library), and Support Vector Classification ($\text {SVC}$) [15] yield the best performance in terms of multiple different metrics for one experiment. Therefore, Kong et al. results give further evidence that CatBoost can outperform other $\text {ML}$ algorithms on categorical, heterogeneous data, whereas Adamovic et al. results, that we cover next, show CatBoost falls behind other techniques when homogeneous, numeric data is used.

Adamovic et al. study is titled “An efficient novel approach for iris recognition based on stylometric features and machine learning techniques.” [23] Stylometry is the study of identifying an author based on the content of his or her work. In their study, stylometry is applied to the Base-64 encoding of iris images as though it were prose, so that identifying the hypothetical author equates to identifying the owner of the iris. For an example of a stylometric feature, from [23][Tab. 1], we see, “The number of vowels in a text.” We consider Adamovic et al. study of properties of the human iris to be a medical study, for the purpose of biometric identification applications.

Their study’s data processing pipeline converts iris images into samples with all numeric features. They use the Chinese Academy of Sciences Institute of Automation ($\text {CASIA}$) Iris Image Database Version 4 as the source of images to fit several $\text {ML}$ models. The models used are as follows: from the Weka system, 1R rule learning [69], C4.5 Decision Tree [70], $\text {SVM}$ [71] and Multiboost [72]. Within the Weka program, these models are named OneR, J48, SMO and MultiboostAB, respectively. Another model used is a version of Random Forest [73] from the R package randomForest. From the Python Scikit-learn library, they use the $\text {SVC}$ algorithm. Finally, they also include CatBoost as a model. Adamovic *et al.* report that for handling class imbalance in their experiments, they use $\text {SMOTE}$ [63], and Majority Weighted Minority Over-sampling Technique ($\text {MWMOTE}$) [74]. The exact nature of class imbalance is not clear in their study. Adamovic et al. write that their data has two classes, Class Y, and Class N. Furthermore, Class Y is the class “the same iris” and Class N is “irises of different persons.” Class Y has 450 samples, and Class N has 2,415 samples. The link to the CASIA dataset^4^ containing the iris data is provided, but at the time of this writing, this site is not accessible. Adamovic et al. write that they use the Recursive Feature Elimination ($\text {RFE}$) [75] and Regularized Random Forest ($\text {RRF}$) [76] methods for feature extraction. They conduct experiments with a 16 feature dataset, derived with RFE, and an 8 feature dataset they obtain with RRF. In addition, they report conducting experiments with all 62 features. The results Adamovic et al. report are very strong in terms of accuracy, precision, recall, F1 score and $\text {AUC}$, regardless of which feature selection technique they use, or which machine learning algorithm they use. All scores they report are close to the maximum values possible, as can be seen in Table 16 that we copy from [23]. We interpret the results in Table 16 to mean that the stylometric data they derive from iris images is so distinctive that the number of features, and ML classifier they use is not very important. There are clear winners in Table 16, but margins are close. Another conclusion we draw from the results is that CatBoost yields weaker performance than other classifiers because the data is derived from a homogeneous source, and in [2], Prokhorenkova et al. mention that CatBoost may not perform as well on homogeneous data as other ML algorithms.

**Table 16:** From [23] original caption, “Iris recognition performances on the CASIA dataset, with the cross-validation performed after the over-sampling (SMOTE).”

Method	Accuracy	Precision	Recall	F1	AUC
All features					
OneR	0.9982 ± 0.003	1.00 ± 0.01	0.99 ± 0.01	0.99 ± 0.01	1.00 ± 0.01
J48	0.9926 ± 0.006	0.99 ± 0.02	0.96 ± 0.04	0.98 ± 0.02	0.98 ± 0.02
SMO	0.9927 ± 0.005	0.99 ± 0.02	0.96 ± 0.03	0.98 ± 0.02	0.98 ± 0.01
SVC	0.9955 ± 0.004	0.97 ± 0.03	1.00 ± 0.01	0.98 ± 0.02	0.99 ± 0.00
RandomForest	0.9980 ± 0.003	1.00 ± 0.01	0.99 ± 0.02	0.99 ± 0.01	1.00 ± 0.00
MultiboostAB	*0.9998* ± *0.001*	*1.00* ± *0.00*	*1.00* ± *0.00*	*1.00* ± *0.00*	*1.00* ± *0.00*
CatBoost	0.9993 ± 0.001	1.00 ± 0.01	1.00 ± 0.00	1.00 ± 0.00	0.99 ± 0.00
RFE-16					
OneR	0.9978 ± 0.003	1.00 ± 0.01	0.99 ± 0.02	0.99 ± 0.01	0.99 ± 0.01
J48	0.9947 ± 0.005	0.99 ± 0.01	0.97 ± 0.03	0.98 ± 0.02	0.99 ± 0.01
SMO	0.9966 ± 0.004	0.99 ± 0.01	0.98 ± 0.02	0.99 ± 0.01	0.99 ± 0.01
SVC	0.9951 ± 0.002	0.97 ± 0.02	0.99 ± 0.01	0.98 ± 0.01	0.99 ± 0.00
RandomForest	0.9983 ± 0.002	1.00 ± 0.01	0.99 ± 0.01	0.99 ± 0.01	1.00 ± 0.00
MultiboostAB	*0.9988* ± *0.002*	*1.00* ± *0.01*	*0.99* ± *0.01*	*1.00* ± *0.01*	*1.00* ± *0.00*
CatBoost	0.9979 ± 0.002	0.99 ± 0.01	1.00 ± 0.01	0.99 ± 0.01	0.99 ± 0.00
RRF-8					
OneR	0.9971 ± 0.003	1.00 ± 0.01	0.98 ± 0.02	0.99 ± 0.01	0.99 ± 0.01
J48	0.9960 ± 0.004	1.00 ± 0.01	0.98 ± 0.02	0.99 ± 0.01	0.99 ± 0.01
SMO	*0.9995* ± *0.002*	*1.00* ± *0.01*	*1.00* ± *0.00*	*1.00* ± *0.00*	*1.00* ± *0.00*
SVC	*0.9997* ± *0.001*	*1.00* ± *0.00*	*1.00* ± *0.01*	*1.00* ± *0.00*	*0.99* ± *0.00*
RandomForest	0.9982 ± 0.003	1.00 ± 0.01	0.99 ± 0.01	0.99 ± 0.01	1.00 ± 0.00
MultiboostAB	0.9977 ± 0.003	1.00 ± 0.01	0.99 ± 0.02	0.99 ± 0.01	1.00 ± 0.00
CatBoost	0.9986 ± 0.002	0.99 ± 0.01	1.00 ± 0.01	1.00 ± 0.01	0.99 ± 0.00

**Fig. 3:**
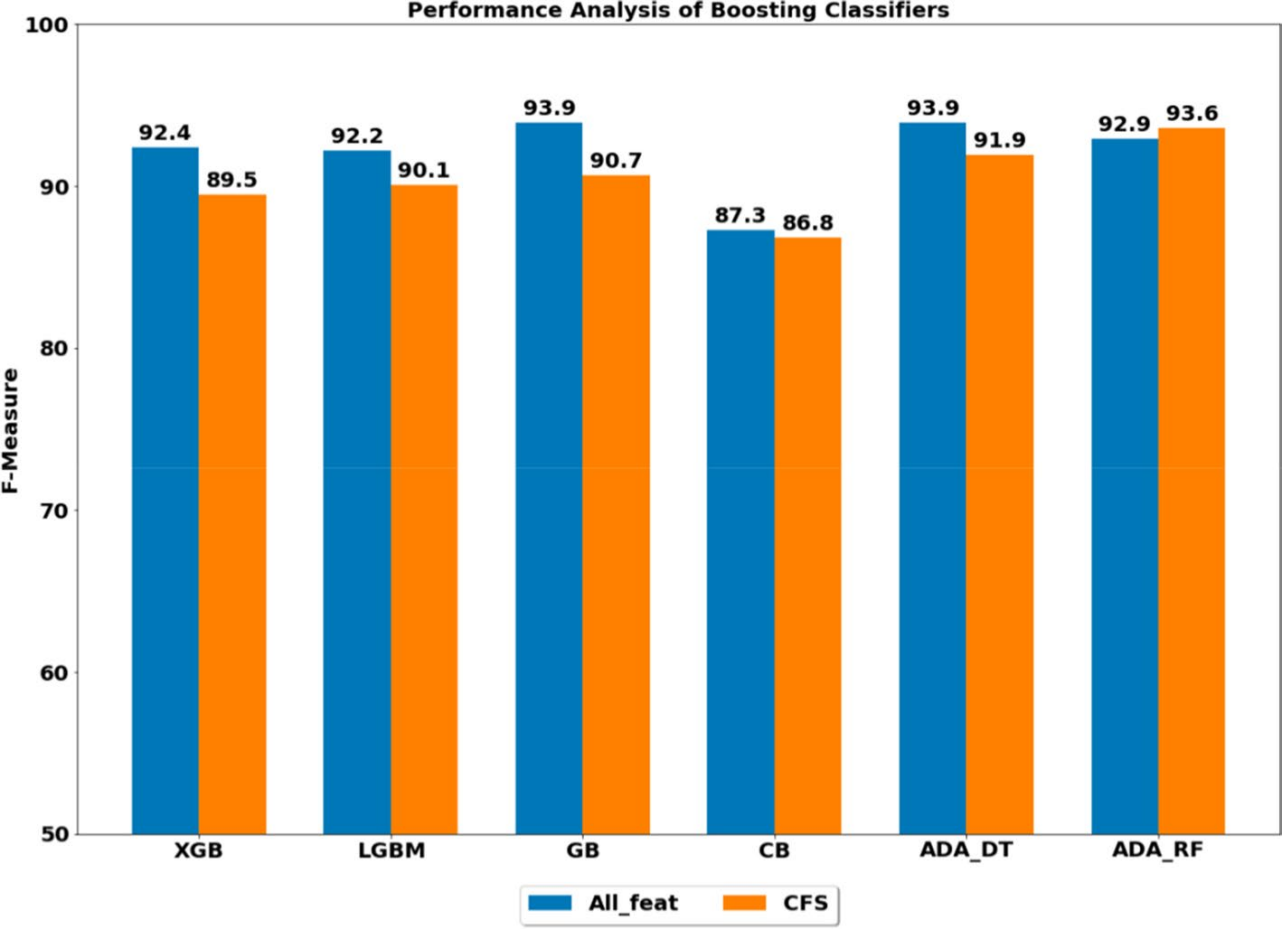
Image from [25] showing relatively weak performance of CatBoost (CB) as compared to XGBoost (XGB), LightGBM (LGBM), Gradient Boosting(GB), AdaBoost using Decision Trees (ADA_DT) and AdaBoost using Random Forest (ADA_RF)

Another example where CatBoost does not appear to do well when pitted against other Gradient Boosted Decision Tree classifiers for $\text {ML}$ tasks where no categorical data is involved is “Performance analysis of boosting classifiers in recognizing activities of daily living”, by Rahman et al. [25]. In their study, the authors compare CatBoost to XGBoost, LightGBM, AdaBoost, and Gradient Boosting [77]. The study documents the performance of these classifiers’ ability to categorize accelerometer and gyroscope sensor data into physical activities of the person using a smart phone. Figure 3 illustrates the relatively weak performance of CatBoost in comparison to other Gradient Boosted Decision Tree algorithms for this $\text {ML}$ task. The legend in Fig. 3 explains the two values for F-measure Rahman et al. report each classifier. They report one F-measure score for models trained with all features, and one F-measure score for models trained with “Correlation-based features” ($\text {CFS}$) [25] [p. 7]. $\text {CFS}$ is a technique for selecting a subset of features that are not correlated with one and other. What stands out to us about this study is that the data the authors use is purely numerical. The data is homogeneous in the sense that it is related to motion since it is from accelerometers and gyroscopes. Rahman et al. study is evidence one should avoid CatBoost for these types of data.

In these studies related to medicine, we find one study that supports the idea that CatBoost is a good choice to use when data is heterogeneous and categorical. That study is Kong et al. [24], where the results for predicting fragility fractures show CatBoost yields the best performance. One reason for this may be that the dataset is heterogeneous data from surveys that cohort members submit for the study. However, we find another study where the data is categorical and heterogeneous in Yang and Bath [26], where CatBoost does not yield the best performance. There, the performance of CatBoost’s competitors are very close and there may not be a statistically significant difference between them. Finally, we see two studies that support the idea that CatBoost is not the best choice for a classifier for datasets with homogeneous features. In the case of Adamovic et al. even though they use feature extraction from raw image data, CatBoost does not outperform other classifiers. We suspect this is due to the original homogeneous nature of the image data they use. In the case of Rahman et al. the data they use is numerical accelerometer and gyroscope data. For $\text {ML}$ applications in Medicine or other fields, CatBoost appears to be more suitable for data that is tabular, such as patient demographic data, or survey data. In the next section, we look at how researchers use CatBoost to make advances in the field of electrical utilities fraud detection.

### Electrical utilities fraud

Coma and Carmona use $\text {ML}$ techniques for electricity theft detection [29]. In their study the authors report using CatBoost, XGBoost, and LightGBM. The subject of the study is interesting to researchers working with Big Data since it involves applying $\text {ML}$ to detect patterns of fraudulent electricity consumption in large-scale data.

In their study, Coma and Carmona work with data that describes millions of a utilities company’s customers throughout Spain. The authors’ goal is to use $\text {ML}$ algorithms to detect non-technical loss in an electricity providers’ service. Non-technical loss ($\text {NTL}$) is a loss due to some abnormality in the place where the provider delivers electricity, or fraud. The proposed advantage of using $\text {ML}$ is to save human time and effort in both finding and correcting the causes of abnormal electricity consumption patterns. The authors do not give specific values of metrics they use to detect non-technical loss. They write that they use GBDT algorithms CatBoost, LightGBM, and XGBoost, but they do not supply a detailed comparison of the performance of each algorithm. They report, “In terms of accuracy, the system succeeds in the detection of NTL. In customers without contract, we have achieved very good results, with campaigns higher than 50% of accuracy. In campaigns to detect NTL in customers with contract, the system has reached up to 36% of accuracy.” This is the greatest detail we find on quantitative results on classifier performance in their study.

Another issue we find in this work is that the authors report they discard AUC as a metric for validating models in favor of the Precision-Recall curve. We have no issue with discarding AUC over Precision-Recall curve. Our issue is that Coma and Carmona are inconsistent. The only quantitative data on performance that we find Coma and Carmona reporting in [29] is in our previous quotation of them where they write about the accuracy of their system in terms of $\text {AUC}$. While their study documents an interesting application of CatBoost to a Big Data problem, Coma and Carmona do not provide enough detail for one to draw a conclusion as to which of CatBoost, XGBoost, or LightGBM served them best. In their study the authors list several reasons why they choose GBDT algorithms over other classification techniques. Their study appears to be a report on a work in progress. It is interesting to researchers working in Big Data since the study involves a large database with data on millions of customers. The conclusion we draw from this study is that CatBoost and other popular GBDT algorithms are being researched by utilities companies as a means to do fraud detection. The next work we cover is on the same subject, and provides more detail on learners the authors use.

Another study that provides more detail on the performance of GBDT algorithms for $\text {NTL}$ detection in the electricity utilities industry is, “Energy theft detection using gradient boosting theft detector with feature engineering-based preprocessing” by Punmiya and Choe [31]. Both [31] and [29] have May 2019 publication dates, which indicates that, for the study of $\text {NTL}$ detection in electricity utilities, there is a trend of using GBDT algorithms.

**Fig. 4:**
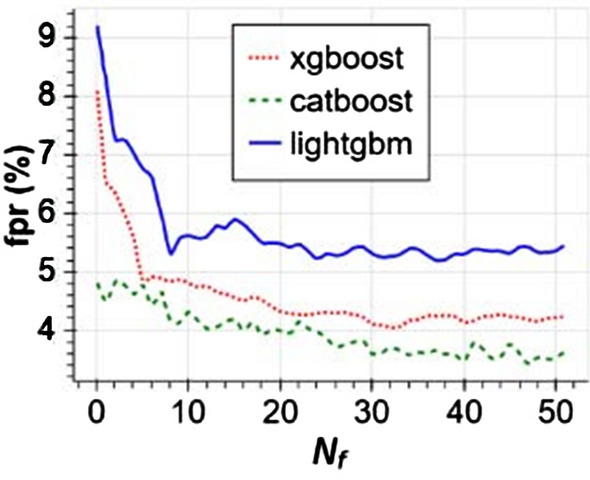
From [31]; false positive rates for XGBoost, CatBoost, and LightGBM as number of features used increases

**Fig. 5:**
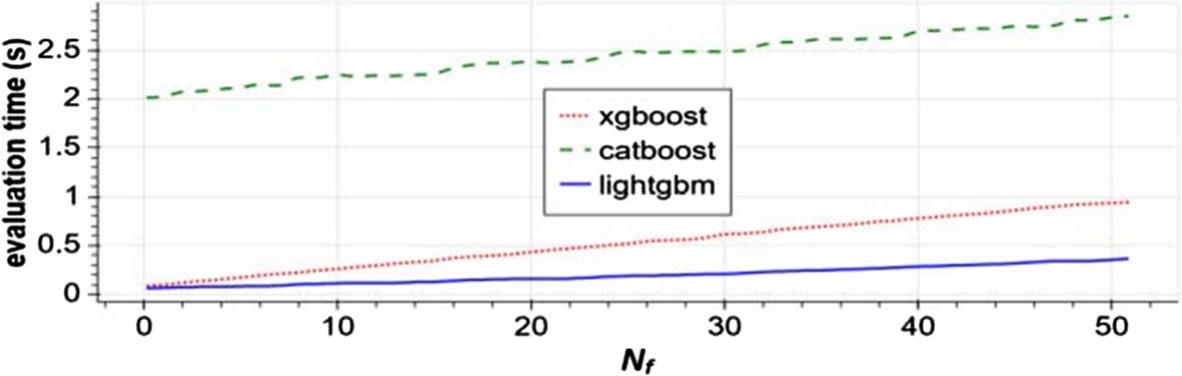
From [31]; Evaluation (total train and test) time for XGBoost, CatBoost, and LightGBM as number of features increases, average of 100 random customers; shows improvement in CatBoost false positive rate when all summary statistics are employed

An important proposal in Punmiya and Choe’s study is a technique for generating samples synthetically. The positive class in their dataset is extremely sparse. Therefore, motivated by a recent study by Buzau et al. [78], that shows the strong performance of XGBoost in $\text {NTL}$, Punmiya and Choe compare XGBoost, CatBoost, and LightGBM to an existing consumption pattern-based electricity theft detector ($\text {CPBETD}$) that is based on Support Vector Machine ($\text {SVM}$). They compare the performance of these classifiers to detect patterns of theft in electricity usage data from smart grid electricity meters. Punmiya and Choe rely on random number generators to generate values in ranges above those in typical non-theft data. In addition, they test the utility of deriving features based on summary statistics (minimum, maximum, mean, and standard deviation) of daily electricity usage. This is noteworthy because we see the same technique used in previous research by Bauder et al. in the similar task of health insurance fraud detection at Big-Data scale, where they add summary statistics based on treatments and procedures aggregated by healthcare provider and year [79]. Like Punmiya and Choe, Bauder et al. work with an imbalanced dataset. The efficacy of including summary statistics may only be specific to Punmiya et al. and Bauder *et al.*’s experiments. There is an opportunity for future research to determine how adding summary statistics may improve a classifier’s performance. Punmiya and Choe find that Gradient Boosted Decision Tree algorithms provide lower false positive and higher true positive rates in their experiments when compared with $\text {CPBETD}$. We interpret the results Punmiya and Choe report as suggestions for handling imbalanced data for use with Gradient Boosted Decision Tree algorithms such as CatBoost. The suggestions are: generate positive class samples synthetically by generating fake observations with features that have values in ranges that could only belong to the positive class if such a sample did exist, and augment features with summary statistics of other features. Punmiya and Choe show the lower false positive rates and higher true negative rates when they employ summary statistics in a table of results we include in Table 17. They also show that CatBoost has the lowest false positive rate, but the longest total train and test time, in the charts that we include copies of in Figs. 4 and 5, respectively. In Punmiya and Choe’s work, we find techniques that are useful for fraud detection that are applicable at Big Data scales. As we shall see in the next work, we cover more applications of GBDT techniques for $\text {NTL}$ detection in the electrical utilities industry.

**Table 17:** From [31], “Performance comparison without or with new feature(s) (average of 100 random customers), where revised theft cases are used

XGBoost	w/o	w/	w/	w/	w/	w/
	Synth	Mean	Std	Min	Max	All 4
DR(%)	94	95	95	95	95	96
FPR(%)	6	5	4	4	4	4
CatBoost	w/o	w/	w/	w/	w/	w/
	Synth	Mean	Std	Min	Max	All 4
DR(%)	97	97	97	97	97	97
FPR(%)	5	6	5	5	5	3
Light	w/o	w/	w/	w/	w/	w/
GBM	Synth	Mean	Std	Min	Max	All 4
DR(%)	97	97	97	97	97	97
FPR(%)	7	7	6	5	6	5

“ Here”, “synth” refers to features derived from summary statistics of daily usage; DR refers to detection rate, or true positive rate, and FPR refers to false positive rate

A third study on $\text {NTL}$ in electrical utilities is “Impact of feature selection on non-technical loss detection” by Ghori et al. [30]. In their study, they propose the Incremental Feature Selection ($\text {IFS}$) technique for $\text {NTL}$ detection. For details on $\text {IFS}$ please see [30, Algorithm 1]. Ghori et al. do not compare $\text {IFS}$ to other feature selection techniques, so its relative efficacy remains to be seen. Hence, a study to evaluate feature selection techniques for classifiers on the task of $\text {NTL}$ is an opportunity for future research.

In their study, Ghori et al. document experiments where they compare CatBoost, Decision Tree ($\text {DT}$), and K-Nearest neighbors ($\text {KNN}$) on the task of detecting $\text {NTL}$ in a specific dataset that they apply $\text {IFS}$ to. The dataset they use consists of electricity consumption information from 80,244 customers over 15 months, with 112 features total. Of the 112 features, they find that only 71 are useful. Ghori et al. report that in their experiments CatBoost outperforms KNN and DT for a full feature set of 71 features in terms of precision, recall and F-measure. CatBoost also outperforms KNN and DT in terms of precision, recall, and F-measure with a reduced set of 9 features obtained with $\text {IFS}$, except for KNN, which has a higher recall than CatBoost for the reduced feature set. They also point out that, as one would expect, CatBoost has a lower training time when they use a dataset with 9 features. As we see in Table 18, CatBoost’s performance suffers with the reduced dataset, but one can use $\text {IFS}$ to trade some performance for training time.

Ghori et al. do not use any technique to balance the class ratio in their data, but they ensure their training and test dataset have the same class ratio. The class imbalance ratio is 96% class, and 4% positive. We include a copy of a table of results from [30] that shows the relative performance of CatBoost, $\text {DT}$ and $\text {KNN}$ with the number of features (9 or 71) as a factor in Table 18.

**Table 18:** From [30], “Precision , recall and F-measure of CatBoost, Decision Tree classifier and KNN for 9 and 71 features”

	Features	CatBoost (%)	DT (%)	KNN (%)
Precision	71	98.11	97.23	94.18
	9	97.40	96.8	96.58
Recall	71	99.27	97.80	45.10
	9	98.68	98.24	99.12
F-Measure	71	98.69	97.51	61.00
	9	98.04	97.53	97.83

Ghori et al. study [30] was published as a part of the 6th International Conference on Data Science and Machine Learning Applications. Apparently they were expanding this work for “Performance Analysis of Different Types of Machine Learning Classifiers for Non-Technical Loss Detection” [52] that appeared in the Journal “IEEE Access” around the same time that [30] was published. Both studies have the same authors, and the data descriptions in both studies indicate they use similar data. In [52], the authors augment the collection of classifiers they use to include an Artificial Neural Network ($\text {ANN}$). Interestingly, we find a description of a process similar to $\text {IFS}$ in [52], but it is not referred to as such. However, in [52] the authors report finding 14 useful features after applying feature selection, whereas in [30] the authors report finding 9 useful features. The results in [52, Tab. 5], comparing the performance of classifiers used in the study, show that CatBoost outperforms other learners in terms of precision and F-measure, but not recall. The same table shows that the algorithm that yeilds the best performance in terms of recall is $\text {ANN}$. However, Ghori et al. list all features of their dataset in [52] [Tab. 6]. We find at least three features, “Type-Premise”, “Type-Bill” and “Type-Consumer” that are categorical. They have definitions “House hold type like house, flats, market etc.”, “Type of bill” and “Connection type”, respectively. Moreover, in [52] [Tab. 7] the authors give lists of hyper-parameters they use, but they do not indicate they set CatBoost’s “cat_features” hyper-parameter. Therefore we cannot conclude that Ghori et al. took maximum advantage of CatBoost’s Ordered Target Statistics encoding for all categorical features in their dataset.

Our search for studies involving CatBoost reveals that researchers are interested in using it for $\text {NTL}$ detection in electrical utilities markets. Coma and Carmona’s contribution of using the SHAP importance for selecting features in their dataset appears to be an earlier publication of an idea that Ghori et al. refine with $\text {IFS}$. Furthermore Ghori et al. expand their investigation into $\text {NTL}$, showing best results for CatBoost in terms of precision and F-measure, but not recall. Punmiya and Choe make a contribution by showing the performance of CatBoost on imbalanced datasets can be improved with the introduction of synthetic data, and the addition of new features from summary statistics of existing features. Ghori et al. propose $\text {IFS}$ for feature selection, but do not compare it against other feature selection techniques. Their results of their classifiers’ performance in terms of accuracy and running time show that one may use $\text {IFS}$ to tune feature set size to trade performance in terms of precision, recall, or F-measure for performance in terms of computation time. These studies show CatBoost is a useful tool for $\text {NTL}$ detection in the electrical utilities industry, with possible applications in fraud detection in other markets. Given the success of other $\text {DT}$ based classifiers for fraud detection [80–82], we see opportunities for applying CatBoost to more fraud detection tasks. In the next section, we take a look at how researchers are using CatBoost to study the weather.

### Meteorology

The first meteorology-related work we cover in the database is “Short-term weather forecast based on wavelet denoising and CatBoost” by Diao et al. [51]. In this study the authors cite the inaugural CatBoost paper [2], by Prokhorenkova et al. as their justification that CatBoost is the superior boosted Decision Tree algorithm. They also mention training times for neural network based solutions are longer than Decision Tree based solutions. This is another motivation Diao et al. have for trying CatBoost. The data that the authors use in the study initially has 38 features. Before feeding the data into CatBoost for regression, the authors apply feature selection by Recursive Feature Elimination ($\text {RFE}$), thus eliminating features with low correlation to the target value, then use some unspecified combination of the feature importance ranking functions built into CatBoost and XGBoost. The final group of features the authors use is not clear in their publication. They provide a table of the least important features, but it is not obvious what the features listed in the table have to do with the features they use in the study. The table is entitled “The Least Important Features,” so one surmises that perhaps they are eliminated during feature selection. After employing these three initial feature selection techniques, Diao et al. write that they use spatio-temporal feature extraction to enhance qualitative features such as temperature, humidity, and wind speed. However, they only discuss a technique for transforming temporal features: month, day of the month and hour, to two-dimensional values, so what we can surmise from the information they provide is that they only perform temporal feature extraction. Diao et al. provide a fair amount of detail on the Wavelet Denoising technique they use to smooth values in the features they select. For results, Diao et al. report scores that are calculated based on root mean square error ($\text {RSME}$) of values they forecast, and the RMAPS-based $\text {RMSE}$ that is not clearly defined. Diao et al. mention they use data from the “weather forecast track in 2018 AI Challenger Global AI Contest” but we do not find a reference for that contest to find out more about RMAPS-based $\text {RSME}$. The formula they give for a score is:

9 $$\begin{aligned} \text {Score} = \frac{RSME\left( \text {RMAPS}\right) - RSME\left( \text {forecast}\right) }{RSME\left( \text {RMAPS}\right) } \end{aligned}$$

We give Diao et al. the benefit of the doubt and assume that the scores they report for models are such that higher scores indicate lower $\text {RMSE}$ of their forecast. In the results Diao et al. report, CatBoost outperforms Random Forest [73], Long Short-term Memory ($\text {LSTM}$) [83], and Seq2Seq [84]. We include this study in our survey since it shows CatBoost may work well for regression tasks when used in conjunction with Wavelet Denoising.

“Evaluation of catboost method for prediction of reference evapotranspiration in humid regions” by Huang et al.is a more robust study on meteorological applications of CatBoost [33]. The principal aim of the study is to use $\text {ML}$ regression models to forecast water evapotranspiration ($\text {ET}$). Huang et al. define $\text {ET}$ as, “The loss of water from the ground and vegetation into the atmosphere, composed of evaporation from ground and vegetation surfaces plus transpiration through vegetation.” Accurate estimates of $\text {ET}$ are important for water resource planning. Huang et al. report on the performance, in terms of $\text {RMSE}$, mean absolute percentage error ($\text {MAPE}$) [85], MBE, and $R^2$ [86] of CatBoost, Vector Machine ($\text {SVM}$) [87] and Random Forest ($\text {RF}$) [73]. The error of the regression models is calculated relative to a reference value of $\text {ET}$, which they provide a formula for. In their experiments, the authors use data from 12 weather stations in Southeastern China. The raw data from the weather stations contains 5 features: daily solar radiation ($R_S$), maximum ($T_{max}$) and minimum ($T_{min}$) air temperatures at 2 m height, relative humidity ($H_r$), and wind speed (*U*) at 2 m height. As part of their experimental design the authors test performance of models with all 5 features, and 7 different subsets of the 5 features. Huang et al. do not report if they employ any well-known techniques to determine feature importance. We note what appears to be a minor typographical error in the definition of $\text {MAPE}$ that Huang et al. supply:

10 $$\begin{aligned} \text {MAPE} =\frac{1}{n}\sum _{i=1}^n \frac{Y_{i,m}-Y_{i,e}}{Y_{i,m}} \end{aligned}$$

According to de Myttenaere et al. definition of absolute percentage error [85], in Huang et al. notation the definition of MAPE should be:

11 $$\begin{aligned} \text {MAPE} =\frac{1}{n}\sum _{i=1}^n \frac{\left| Y_{i,m}-Y_{i,e}\right| }{\left| Y_{i,m}\right| } \end{aligned}$$

where $Y_{i,e}$ is the estimated value and $Y_{i,m}$ is the measured (actual) value. According to Google Scholar, at the time of this writing, de Myttenaere et al. [85] has 185 references. This lends credibility to the claim that their definition of absolute percentage error is widely accepted. We do not find that Huang et al. provide a citation for their definition of MAPE, so we cannot validate whether it is widely accepted, and therefore conclude it is a typographical error. Otherwise, one may choose to ignore results on performance in terms of MAPE for the regression models. Huang et al. also do not provide a reference or definition of the acronym MBE, but they provide a formula for it. This formula indicates MBE is simply the mean value of the difference of estimated and measured values:

12 $$\begin{aligned} \text {MBE}=\frac{1}{n}\sum _{i=1}^n \left( Y_{i,m} - Y_{i,e}\right) \end{aligned}$$

**Fig. 6:**
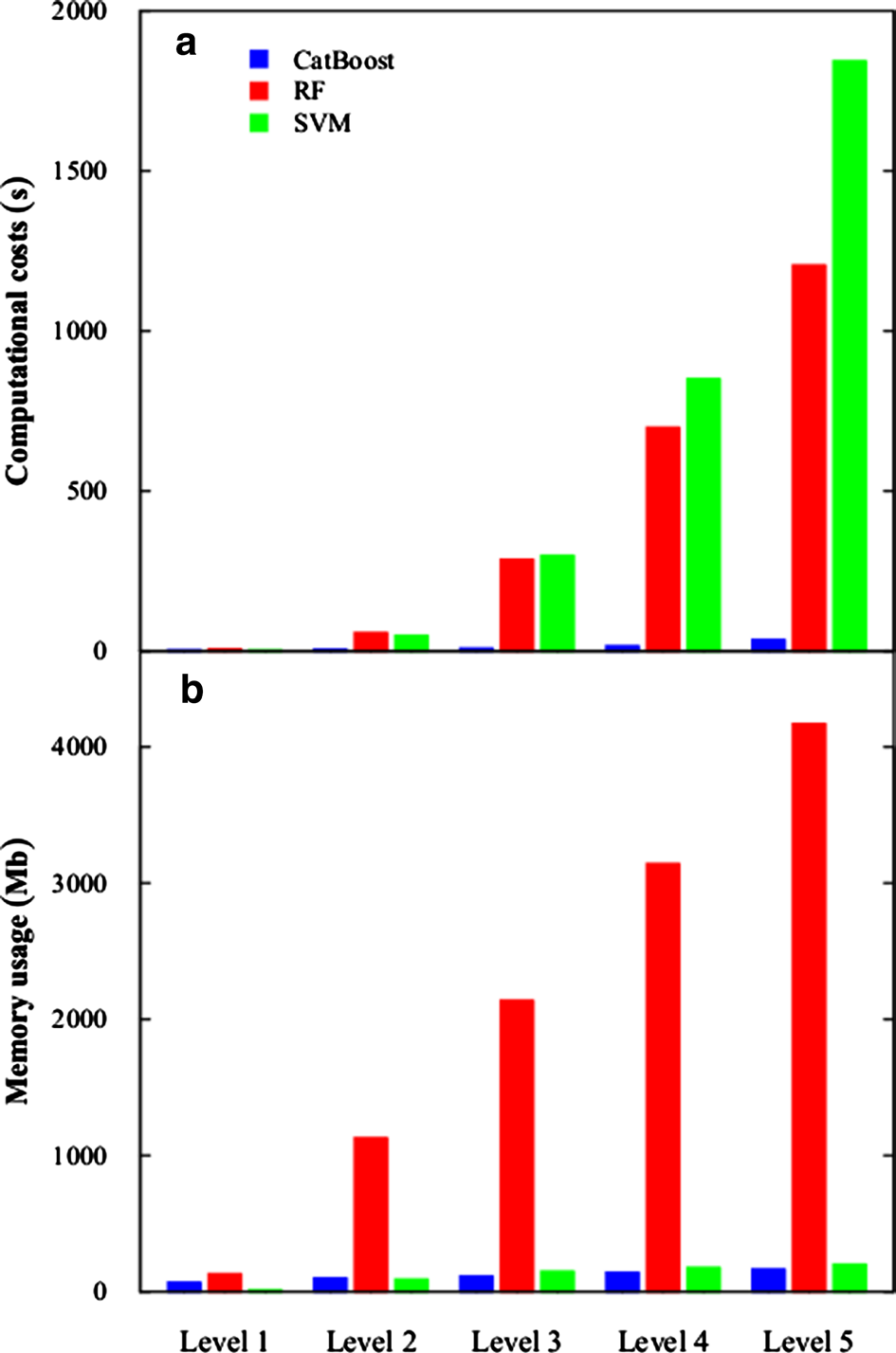
Image from [33] illustrating the relative efficiency of CatBoost, according to Huang et al. Level 1 is 10 years’ data from a single station, Level 2 is 10 years’ data from 12 stations, Level 3 is 20 years’ data from 12 stations, Level 4 is 30 years’ data from 12 stations and Level 5 is 40 years’ data from 12 stations

The authors report that CatBoost works best when inputs have no missing values, but that $\text {SVM}$ outperforms CatBoost when inputs have missing values. In the context of their study, the term “missing values” means features removed from their dataset. However, the most interesting result from a Big Data perspective is the comparison that Huang et al. make between the running times and memory usage of CatBoost, $\text {SVM}$ and $\text {RF}$. A copy of bar charts from [33] in Fig. 6 shows CatBoost consumes less time and less memory than its competitors. The time and memory usage reported in Fig. 6 is measured for different levels of data, where the level corresponds to the size of the dataset. If resource consumption is the primary concern, the results in Fig. 6 suggest one should select CatBoost when choosing a Gradient Boosted tree ensemble algorithm. However, we do not see where Huang et al. include hyper-parameter settings for the models they evaluate, so one cannot verify that they did not use settings that would result in unnecessary resource consumption.

Another study related to predicting weather-related phenomena with CatBoost is “Predicting daily diffuse horizontal solar radiation in various climatic regions of China using support vector machine and tree-based soft computing models with local and extrinsic climatic data” [32], by Fan et al. This study examines the performance of different algorithms for predicting the total amount of solar radiation that reaches the Earth. Fan et al. cite previous studies that show $\text {SVM}$ outperforms some neural network based approaches. For more background on neural networks, please see [88]. Fan *et al.* compare several Decision Tree based algorithms to $\text {SVM}$. The conclusion that Fan et al. make in this study is that CatBoost is nearly as accurate as $\text {SVM}$, but has running times that are lower than $\text {SVM}$’s. This confirms Huang *et al.*’s findings we report above. The results of two of their experiments show that CatBoost runs about 1.9 times as fast as $\text {SVM}$. In a third experiment they find that CatBoost is 33.9 times faster than $\text {SVM}$. Therefore, this result is interesting to researchers working with Big Data, since CatBoost could provide a noticeable reduction in processing time for long-running jobs. Another interesting point about the study is that Fan *et al.* report that they used a Grid Search hyper-parameter optimization technique for the learners they compare. This is the second instance where we find CatBoost is reported to be the best performing learner when the authors also report using a hyper-parameter tuning technique. The first instance is in [20] that we cover earlier.

The studies on applications of CatBoost to Meteorology that we find here show two things. The first is that the CatBoost regression variant is a viable candidate for forecasting weather-related data. The data that Huang et al. and Fan *et al.* report on using seems heterogeneous since they are measurements of different types of climatic data. Diao et al. do not provide quite enough detail on their data for us to be certain about its degree of homogeneity. The second is that CatBoost has better running times than $\text {SVM}$ for weather-related regression problems. A comparative complexity analysis of $\text {SVM}$ and CatBoost is outside the scope of this study. Please see Appendix C of [2] for a complexity analysis of CatBoost. Fan et al. may have obtained this finding because they employ a hyper-parameter optimization technique. Next, we cover applications of CatBoost in the field of Psychology.

### Psychology

One example where CatBoost as a classifier outperforms other classifiers is “Screening of anxiety and depression among the seafarers using machine learning technology” by Sau and Bhakta [35]. In this study CatBoost outperforms Random Forest, Logistic Regression, Naïve Bayes, and Support Vector Machine classifiers in the task of identifying individuals that suffer from anxiety or depression. It is important to note that the raw data the authors use in this study is from a survey where they treat the answers to the survey as features. Eight out of fourteen of the features from the survey are categorical values. This helps explain why CatBoost is the best performing classifier for the machine learning task in [35]. The authors write, “Five machine learning classifiers (Logistic Regression, Naïve Bayes, Random Forest, Support Vector Machine and Catboost), those [*sic*]can handle the binary outcome variable (labels) with mixture of categorical and continuous features, are selected for comparison purpose.” So, it appears that the authors take advantage of built-in support for encoding categorical features in all the learners they use. These results may be an indication that CatBoost has better support for encoding categorical features. The authors also report hyper-parameter settings they use for each learner. This could make the study a good starting point for research into CatBoost’s sensitivity to hyper-parameter settings since the settings are documented.

Another study in the Psychology domain that uses CatBoost is “Machine learning identifies the dynamics and influencing factors in an auditory category learning experiment” [34], by Abolfazli et al. The study is an investigation into the ability of humans to categorize sounds. Hence, experiments in the study involve playing sounds for the subjects and asking them to identify which category the sound belongs to. The authors list three specific goals in the study: (1) gain insight into how humans learn to categorize sounds, (2) detect when subjects are near the limit of their capacity to learn new categories of sounds and (3) determine whether subjects will be able to learn a new category of sound. Abolfazli et al. state that their motivation for the third goal is to prevent fatigue in test subjects. Their study is fascinating because it is an example of how to use machine learning to better understand human learning. However, we see an opportunity for further research since the authors do not compare the performance of CatBoost to other $\text {ML}$ algorithms on their $\text {ML}$ task. The task in Abolfazli et al. experiments is to estimate whether a person will learn to classify types of sounds given the person’s performance so far.

The technique Abolfazli et al. use for feature engineering is interesting because it could be part of an experimental design for other experiments with the goal of predicting humans’ ability to learn. They measure the true positive rate and false positive rate of the test subjects’ classifications of sounds in blocks of 40 trials, and use these scores as features for a CatBoost $\text {ML}$ model. Please see [34] [Tab. 1] for more details on these features. The subjects’ performance over the duration of the experiment makes a time series. In [34] [Tab. 2] Abolfazli et al. report the progressive increase in the balanced accuracy [89] scores CatBoost yields as they increase the amount of data they use as input to CatBoost. This result shows CatBoost’s ability to predict whether a subject will learn to classify sound categories before the end of the experiment.

The two studies we find involving CatBoost and Psychology cast CatBoost in a positive light. In the first case of Sau and Bhakta [35], the learner does well, perhaps because the data are survey data that are categorical and heterogeneous. However, in the second study by Abolfazli et al. [34], the data are classification error rates of the test subjects, that are acting as classifiers during testing. Therefore, it may be the case that another learner would do better if used in the same experiment. Now we pivot from Psychology to the subject of Traffic Engineering. To get started on that subject we take a look at a study on identifying driving style.

### Traffic Engineering

We find one study related to identifying driving style. This study falls under the category of research with a likely application to self-driving automobiles. One interesting aspect of the study is that it is an application of CatBoost for semi-supervised learning. This study is titled “A semi-supervised tri-catboost method for driving style recognition” by Liu et al. [36]. In this article the authors propose the Tri-CatBoost method for labeling data. This method leverages labeled data to impute labels for unlabeled data. Tri-Catboost employs three CatBoost classifiers that are trained on Bootstrap samples of labeled data. After training, Tri-CatBoost begins an iterative process that involves tentatively labeling data and updating the three models. Liu et al. refer to this as the “minority obeying majority” strategy. The minority obeying majority strategy involves iteratively training one CatBoost classifier on data labeled by other CatBoost classifiers. Iterations continue until the classifiers are in agreement on labels they are generating, and all the classifiers have stable error rates when re-evaluated on the labeled data. An opportunity for future research is in the domain of mitigating class noise. One could employ the Tri-CatBoost method, using samples of labeled data where one is confident that the samples have little to no class noise, and treat samples of data with class noise as unlabeled data. One could then take the resulting labels that Tri-CatBoost imputes for the data with class noise as a dataset cleansed of class noise.

**Fig. 7:**
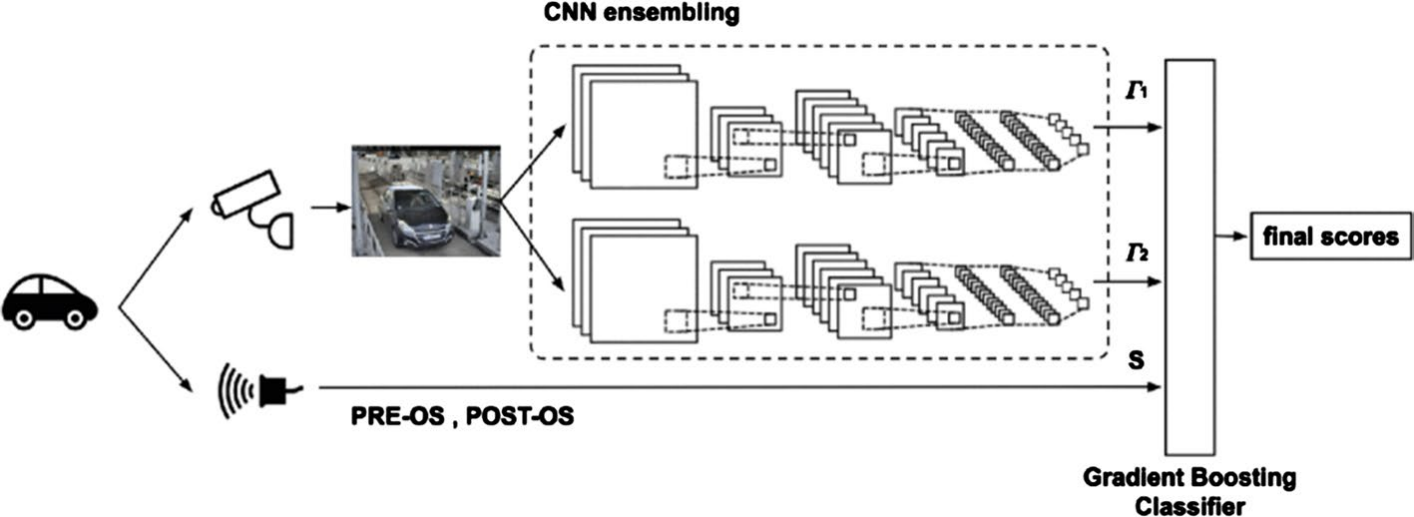
Image from [90] depicting ensemble architecture of system for automatic vehicle detection; CNN and OS output are fed to CatBoost

Another application of CatBoost in the field of Traffic Engineering is “Accurate classification for automatic vehicle-type recognition based on ensemble classifiers” by Shvai et al. [90]. The authors cover an interesting ensemble technique that involves Convolutional Neural Networks ($\text {CNN}$) [91], Optical Sensors ($\text {OS}$), and CatBoost. Shvai *et al.*’s description of OS’s indicates that they are devices that measure the height and number of axles on a vehicle to return a categorical output value for the vehicle type. The output of the CNN and OS are combined into a vector, which becomes the input to CatBoost. We include a copy of the architecture diagram in Fig. 7. Shvai et al. design has the practical application of automated toll collection. The system is an example of how GDBT’s work well with heterogeneous data; since, in their architecture CatBoost takes data of two completely different types. Shvai et al. report that they experimented with substituting different classifiers in place of CatBoost, but they get the best performance in terms of accuracy with the system as depicted in Fig. 7 with CatBoost. The accuracy of this system in the task of vehicle type identification is 99.03, which is an impressive improvement over the performance of OS’s that they report as 52.77.

A third study we find that is related to Traffic Engineering has to do with the movement of people between urban centers. In “Reconstructing commuters network using machine learning and urban indicators” [7], Spadon et al. report that they choose XGBoost over CatBoost for their study because CatBoost and XGBoost have nearly equivalent performance in terms of accuracy, but XGBoost is about fifty times faster per training iteration than CatBoost. One should not jump to the conclusion that XGBoost is better suited to Big Data problems based on what Spadon et al. report on the relative running times of XGBoost and CatBoost, since Prokhorenkova et al. present opposing results in [2]. Prokhorenkova et al. write that CatBoost’s mean tree construction time is about 4 times faster than XGBoost. One iteration of a $\text {GBDT}$ algorithm is the construction of a Decision Tree, so it would behoove us to know the root cause of the discrepancy in running times that Spadon et al. and Prokhorenkova et al. report. To be clear about Prokhorenkova et al. claims about CatBoost’s and XGBoost’s running time performance, we include a copy of a table from Appendix C.2 of “CatBoost: unbiased boosting with categorical features,” in Table 19. The results in Table 19 are for running CatBoost on the Epsilon dataset, whereas Spadon et al. training time results are on a dataset they derived from Brazilian census data. The Epsilon dataset has 2,000 features whereas the dataset from Spadon et al. appears to have a much larger number of features. It is not clear from Spadon et al. paper exactly how many features their dataset has, but it could be a sparse dataset with thousands of features since they model their data as a graph with 5,565 vertices, and they indicate that the weights of edges in the graph form some features in their dataset. Since there are thousands of vertices, each vertex has thousands of edges incident to it. We know the value of most edge weights is zero since Spadon et al. report that 55,247 of the edges have non-zero weights. In addition, Spadon et al. document that they use 22 additional features for each city from the Brazilian census data. Therefore, the Epsilon dataset, and the dataset from Spadon et al. have a dissimilar number of features. This may account for the difference in training performance that Spadon et al. and Prokhorenkova *et al.* report.

**Table 19:** From [2], showing the mean tree construction time in seconds

	Time per tree
CatBoost Plain	*1.1* s
CatBoost Ordered	1.9 s
XGBoost	3.9 s
LightGBM	*1.1* s

Italic text from the original work, indicates shortest tree construction time

In the field of Traffic Engineering, we see researchers have applied CatBoost to very different problems. However, the first study [36], by Liu et al. is interesting in terms of the new semi-supervised technique presented in it. The second study [90] we cover by Shvai *et al.* shows positive results for CatBoost, and we feel one reason for that is the heterogeneous nature of the data they use in the study. Finally, Spadon et al. [7] apply CatBoost to a graph-related problem, and we find only two such graph-related studies, the other being [38], by Yi et al. While Spadon et al. reject CatBoost for efficiency reasons, it could be worthwhile to investigate whether hyper-parameter optimization would result in improved efficiency for CatBoost. In the next section, we transition from the subject of traffic, to one related to network traffic, and Cyber-security.

### Cyber-security

Detection of computer network attacks is in the domain of Big Data, and fast detection times are important for two reasons. First of all, consumers demand low latency internet service for applications such as gaming, high frequency trading, voice communications, and so on. At the same time, if a system in one’s network is under attack, one would like to know that as soon as possible. Attack detection may introduce latency, so, there is a trade-off between performance and security. One recent study where the authors employ CatBoost to detect attacks is “Attack detection in enterprise networks by machine learning methods” [37], by Bakhareva et al. Here, the authors find that CatBoost outperforms LightGBM, Linear Support Vector Machine Classifier, and Logistic Regression, in terms of cross validation balanced accuracy, balanced accuracy, F1 score, precision, recall, and $\text {AUC}$. However, they also report that CatBoost has longer training and prediction times. The authors report mixed results for the time it takes a trained CatBoost model to make a prediction versus other models. Furthermore, the authors report classification time for the algorithms they compare on entire datasets. In one case, CatBoost is slowest, taking 7.25 seconds to classify instances of the CICIDS [92] dataset as attack/not attack traffic. On the other hand, CatBoost outperforms LightGBM when the authors use CatBoost to classify CICDS into types of attacks. However, in the multi-class case, Support Vector Machine, and Logistic Regression are faster. The results Bakhareva et al. report in [37] show that CatBoost is the best detector of attacks, but there is a trade-off in terms of running time.

We find only one study where researchers use CatBoost in the area of Cyber-security. We see opportunities for future research to apply CatBoost to network security tasks given the success of other researchers in the field [93, 94] and [95]. Therefore, there may be further opportunities to apply CatBoost to other problems where GBDT’s are known to provide good solutions for Cyber-security challenges. CatBoost’s applicability in a wide range of subjects implies that we should be able to find more problems in the realm of Cyber-security where CatBoost is a good solution. Bakhareva et al. results imply that CatBoost is a good choice when accuracy in identifying attacks is more important than latency considerations in designing network security systems. Next, we turn our attention to the subject of CatBoost in Bio-chemistry.

### Bio-chemistry

Yi et al. apply CatBoost in a study on predicting associations between molecules entitled “Construction and analysis of molecular association network by combining behavior representation and node attributes” [38]. In this paper the authors construct a graph with eight types of nodes. The types of nodes are: proteins, micro-ribonucleic acids ($\text {miRNA}$) [96], long non-coding ribonucleic acids ($\text {lncRNA}$) [97], messenger ribonucleic acids ($\text {mRNA}$) [98], circular ribonucleic acids ($\text {circRNA}$) [99], drugs, microbes, and diseases. The edges in the graph are associations between two nodes of different types. In this study, Yi *et al.* call this graph the molecular association network ($\text {MAN}$). Yi et al. use the High Order Proximity preserved Embedding ($\text {HOPE}$) [100] algorithm to learn a vector representation of the nodes in the MAN. They use the components of this vector representation as some features of a dataset for a classifier, and they obtain more features for the dataset from attributes of the different types of nodes in the MAN. However, not all the attributes of the different types of nodes have the same dimensionality. So, they employ an autoencoder to learn representations of the nodes in the MAN such that the representations are vectors all having the same number of components. The components of the vectors that the autoencoder learns constitute more input values for a classifier. The composite of the vector representation that HOPE learns, and the vector representation that the autoencoder learns is the complete representation of a node in the MAN. Though Yi et al. do not explicitly state this, we believe it must be the case that the dataset they use with a classifier consists of pairs of the composite representations of nodes in the MAN, where a pair is labeled as a member of the positive class if there is an association between nodes, and a member of the negative class otherwise.

After Yi et al. obtain the labeled dataset, they compare the performance of several classifiers in their ability to predict associations between members of the MAN. The performance metrics they use are: accuracy, sensitivity, specificity, precision, and Mathews’ correlation coefficient. The classifiers they use are: XGBoost, AdaBoost [101], Random Forest, Logistic Regression, and CatBoost. Table 20 is a copy of [38] [Tab. 3], that shows the results of their performance evaluation. CatBoost has the best results of all classifiers in all metrics except for specificity. The work Yi et al. conduct in this study is interesting because it illustrates a way to use $\text {ML}$ techniques, including CatBoost to work with a heterogeneous network of objects.

**Table 20:** From Yi et al. the proposed method is CatBoost

Method	Accuracy (%)	Sensitivity (%)	Specificity (%)
MAN-HOPE-LR	83.75 ± 0.11	83.21 ± 0.47	84.30 ± 0.32
MAN-HOPE-Ada	84.73 ± 0.18	85.53 ± 0.29	83.93 ± 0.22
MAN-HOPE-RF	92.66 ± 0.12	*92.03* ± *0.15*	93.29 ± 0.22
MAN-HOPE-XGB	89.56 ± 0.41	90.60 ± 0.28	88.51 ± 0.95
Proposed method	*93.30* ± *0.12*	91.50 ± 0.14	*95.10* ± *0.11*

Method	Precision (%)	MCC (%)	AUC (%)
MAN-HOPE-LR	84.13 ± 0.20	67.52 ± 0.22	91.58 ± 0.13
MAN-HOPE-Ada	84.19 ± 0.18	69.48 ± 0.36	92.07 ± 0.13
MAN-HOPE-RF	93.21 ± 0.20	85.33 ± 0.24	97.12 ± 0.05
MAN-HOPE-XGB	88.75 ± 0.81	79.13 ± 0.79	96.02 ± 0.24
Proposed method	*94.91* ± *0.11*	*86.66* ± *0.24*	*97.93* ± *0.08*

Best metrics are highlighted in italic; we split table in two for legibility

One interesting thing to note about Yi et al. study is that it involves a graph representation of data, and experiments with systems where CatBoost is a component, and others where XGBoost is a Component. We see in Table 20 that CatBoost outperforms XGBoost. This is quite different from the results Spadon et al. report on a study involving graphs of data, where the authors report nearly similar performance for both CatBoost and XGBoost. This is evidence that not all problems involving data with graph representations are best solved with a particular GBDT implementation. It also raises further interest in the question of how CatBoost’s sensitivity to hyper-parameters and hyper-parameter tuning impacts CatBoost’s performance in graph-related $\text {ML}$ problems. Next, we cover an application of CatBoost to the study of on-line marketing.

### Marketing

**Fig. 8:**
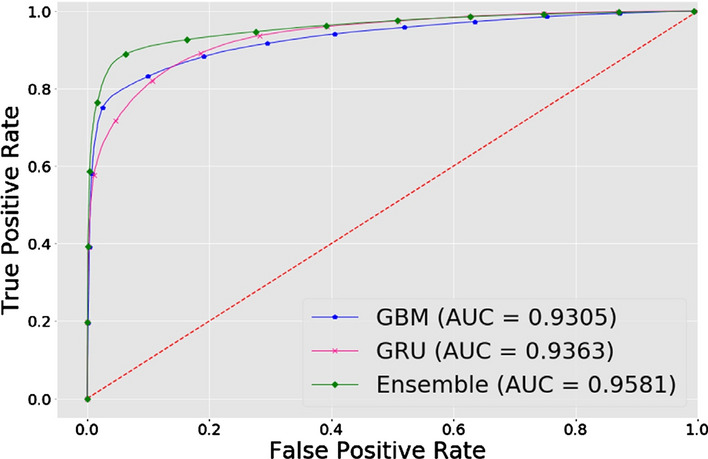
Image from [39] illustrating best results for ensemble of CatBoost and $\text {GRU}$; Here, the authors refer to CatBoost as Gradient Boosted Machine (GBM)

Clickstream data is web application usage data that web applications collect as users interact with the web sites the application provides. Busy web sites with millions of daily users can generate large amounts of clickstream data that falls into the domain of Big Data. “Predicting online shopping behaviour from clickstream data using deep learning” by Koehn et al. is a study on using $\text {ML}$ to predict user behavior from clickstream data [39]. This study focuses largely on neural networks for making these predictions, but CatBoost plays a very important role. The authors find that they get their best results when they employ an ensemble of a Gated Recurrent Unit ($\text {GRU}$) based neural network and CatBoost. The ensemble technique the authors use is to compute the mean value of the output of the neural network and CatBoost for their final prediction. Koehn *et al.* give some important practical advice for choosing to use the ensemble of the $\text {GRU}$-based neural network and CatBoost. The advice is that when one observes two strong models with low correlation between their output values, an ensemble of those two models may perform even better. Therefore, researchers considering taking on work to classify clickstream data may find that CatBoost will form part of an ensemble with the best performance. Figure 8 shows an image reproduced from Koehn et al. [39] (Fig. 8), that demonstrates how the ensemble technique provides the best AUC for predicting user behavior from clickstream data.

**Fig. 9:**
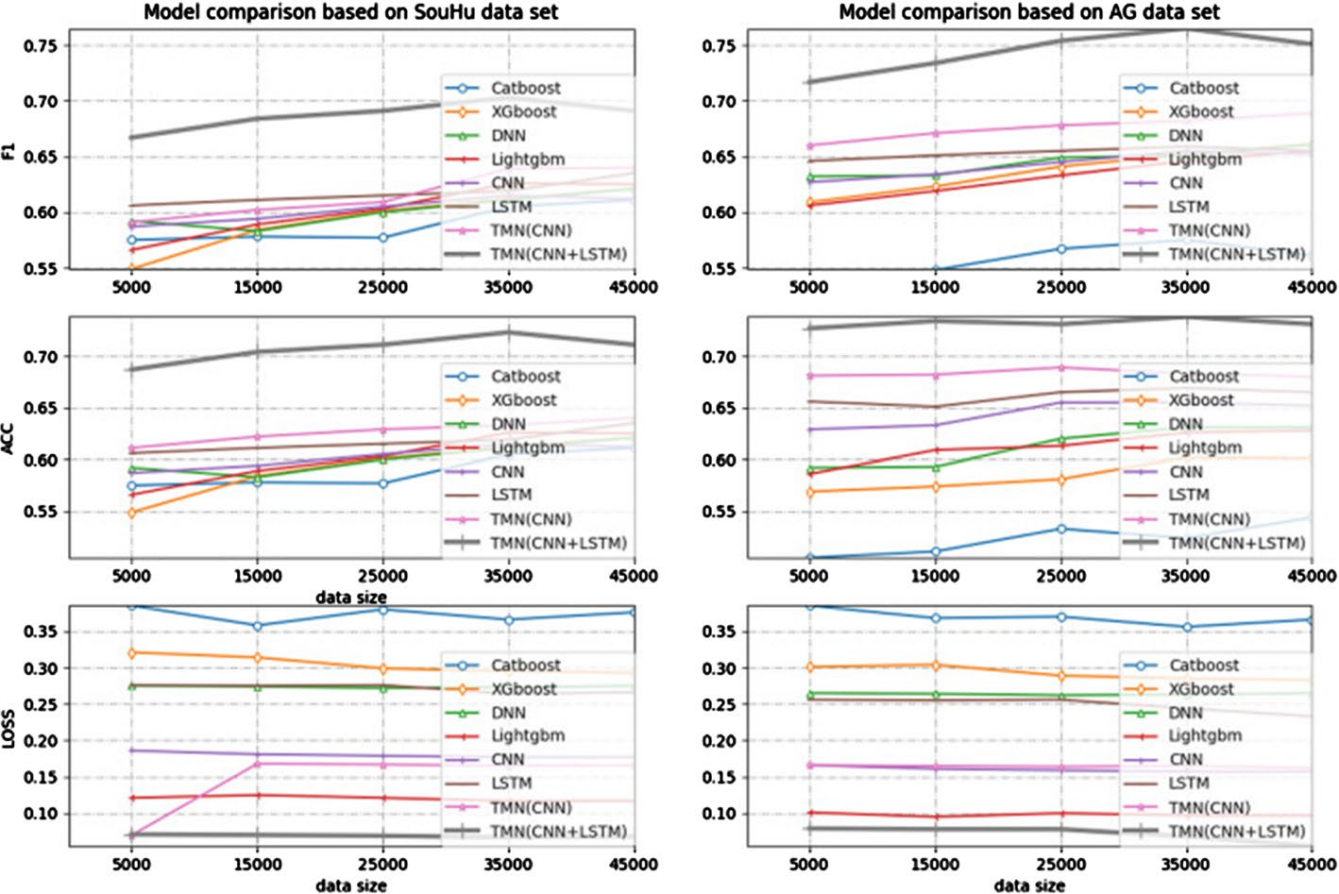
Image from [102] illustrating neural network based algorithms outperforming Gradient Boosted tree algorithms for classifying homogeneous text data in the SoHu dataset of news articles labeled as with or without marketing intent

Another example of a study that shows the inferiority of gradient boosted tree algorithms to neural networks for $\text {ML}$ tasks involving homogeneous data is “A clstm-tmn for marketing intention detection” by Wang et al. [102]. In this study the authors compare various algorithms on the task of classifying text that contains news with marketing intent, and text that contains news without marketing intent. Hence, the $\text {ML}$ task has as input homogeneous data of natural language text. The results that Wang et al. present clearly show CatBoost, LightGBM, and XGBoost under-perform several neural network based algorithms. We include a copy of the key results here in Fig. 9 that provide evidence for Prokhorenkova et al. claim that neural network based solutions may work better than $\text {GBDT}$’s for tasks involving heterogeneous data.

The two studies we find related to Marketing that involve CatBoost serve to point out important considerations to make when deciding to use GBDT’s or neural network based solutions. The first is that an ensemble technique may work well, as in the case of Koehn *et al.* The second is that for homogeneous data, GBDT’s may not be the ideal choice, as is the case with Wang et al. The final subject area we cover is Biology.

### Biology

We find one study related to clear cell renal cell carcinoma ($\text {ccRCC}$), “Ct-based machine learning model to predict the fuhrman nuclear grade of clear cell renal cell carcinoma” [28], by Lin et al. According to The National Cancer Institute [103], $\text {ccRCC}$ is a form of kidney cancer that makes up about 80% of all kidney cancer cases. Therefore, advances in treating $\text {ccRCC}$ stand to benefit the largest number of people who suffer from kidney cancer. In their study, the authors employ CatBoost to classify Magnetic Resonance $\text {MR}$ and Computed Tomography $\text {CT}$ in the task of identifying $\text {ccRCC}$ in the images. Their study does not appear to be research where authors use Big Data techniques. The authors do not report the size of their dataset, but they write that the CT images they use come from a cohort of 231 patients. However, we chose to include this study since image classification for disease diagnoses is potentially a machine learning task where one could employ Big Data techniques. After selecting images for their cohort, Lin et al. indicate that they use the ITK-SNAP^5^ application to segment the images into regions of interest. The ITK-SNAP website mentions that ITK-SNAP application performs “semi-automatic” segmentation. Therefore, future work to apply the results of this study in a Big Data setting, would be to apply a fully automated technique for medical image segmentation, such as those covered in “Automated medical image segmentation techniques” [104], by Sharma and Aggarwal. The remaining components of their system for classifying images are fully automated. Lin et al. use a Python library for extracting features from the segmented images, and then use these features as input to CatBoost. For the classifier component of their system, the authors use CatBoost exclusively. They report encouraging performance metrics, but the results would be more meaningful if they employed other classifiers. One would like to know if any other classifiers, especially a classifier built on a convolutional neural network, would have significantly different performance.

Another work related to the study of diseases, and one study that researchers may find germane in the wake of the Covid-19 pandemic is “Diseases spread prediction in tropical areas by machine learning methods ensembling and spatial analysis techniques” [27], by Kolesnikov et al. In this study the authors use several $\text {ML}$ regression algorithms to predict the number of dengue fever cases in a region during a particular week. One of the learners that the authors employ is CatBoost. CatBoost is not presented as the best performing algorithm in this study. The best performing regression algorithm in this study is a combination of XGBoost and $\text {LSTM}$. The authors do not document whether they examined the performance of other Gradient Boosted Decision Tree ensemble algorithms with $\text {LSTM}$, so there is an opportunity to investigate how a combination of CatBoost and $\text {LSTM}$ would compare to the combination of XGBoost and $\text {LSTM}$. We could see results similar to what Koehn et al. find, where CatBoost is the best choice for an ensemble of a $\text {GBDT}$ and $\text {GRU}$. $\text {GRU}$’s and $\text {LSTM}$’s are both recurrent neural networks. The outcome of that future work would help researchers understand how well-suited CatBoost is for regression problems involving time-series data. Furthermore, we foresee researchers having a stronger interest in studies similar to Kolesnikov et al. due to the Covid-19 pandemic.

In summary, we see CatBoost for two different applications in the field of Biology: one related to detection of types of kidney cancer, and another for predicting the spread of disease. In the case of Lin et al. for detecting kidney cancer from image-based data, we conjecture one might obtain better results employing a classifier more suited to homogeneous data. On the other hand, for studies like the one Kolesnikov et al. conduct, CatBoost may be a good choice for an ensemble technique with some type of recurrent neural network.

## Conclusions

The research we cover in this survey leads us primarily to the conclusion that CatBoost is a good candidate for $\text {ML}$ implementations involving Big Data. Researchers should consider using it with datasets that are heterogeneous, and have categorical features. The results we cover imply one cannot rule out other GBDT implementations for specific problems, and that a common practice is to use more than one. However, since it is easy to use because of its automatic handling of categorical values, and strong performance relative to other GBDT implementations, we believe CatBoost will remain a suitable choice for many applications for some time.

Another aspect of CatBoost we uncovered is its sensitivity to hyper-parameter settings. Settings for the maximum number of iterations for CatBoost to use, the maximum depth of constituent Decision Trees, and the maximum number of combinations of categorical features to combine are values the user can alter to trade resource consumption for performance. Furthermore, values that researchers use for these hyper-parameters may help explain discrepancies in performance of CatBoost with respect to other learners.

Our interdisciplinary approach highlights the wide variety of fields where researchers have employed CatBoost. This shows not only the generality of CatBoost, but also that researchers who have a good understanding of it have an opportunity to collaborate with experts in other fields. We see several opportunities for such collaboration. We find many studies where researchers do not document the use of any hyper-parameter optimization method for the learners they use. This implies we can obtain more insightful results simply by doing that optimization. We see two examples of studies where the authors use model blending, but it is not clear how they derive the coefficients for the outputs of constituent models, so there is an opportunity for research on a robust method for blending model outputs where one of the models is CatBoost. In our coverage of the uses of CatBoost for electricity theft detection, we find opportunities for future research into feature selection techniques. Also, in our review of applications of CatBoost in electricity theft detection, we find a chance for research into the extent of the generality of Punmiya and Choe’s technique of augmenting a dataset with summary statistics to boost performance. Another interesting technique that we find in the domain of Traffic Engineering research is the Tri-CatBoost semi-supervised technique for labeling data that may have applications for problems involving datasets with class noise. Our description of GBDT’s, and the CatBoost implementation, as well as our coverage of various studies, provides expert knowledge that empowers one to employ CatBoost in these or many other future endeavors.

## Data Availability

Not applicable.

## Abbreviations

ADASYN: Adaptive synthetic sampling approach
ANN: Artificial neural network
API: application programming interface
AUC: area under the receiver operating characteristic curve
BDLSTM: Bidirectional long short term memory
BNN: Bagging neural network
CASIA: Chinese Academy of Sciences Institute of Automation
ccRCC: Clear cell renal cell carcinoma
CFS: Correlation-based features
circRNA: Circular ribonucleic acid
CNN: Convolutional neural network
CPBETD: Consumption pattern-based electricity theft detector
CT: Computed tomography
DT: Decision tree
ELSA: English longitudinal study on aging
ESO: European Southern Observatory
ET: Evapotranspiration
GBDT: Gradient bosted decision trees
GRU: Gated Recurrent Unit
HOPE: High order proximity preserved embedding
IFS: Incremental feature selection
KiDS: Kilo Degree Survey
KNN: K-nearest neighbors
lncRNA: Long non-coding ribonucleic acid
LR: Logistic regression
LSTM: Long short-term memory
MAN: Molecular association network
MAPE: Mean absolute percentage error
MCC: Matthews’ Correlation Coefficient
miRNA: Micro-ribonucleic acid
ML: Machine learning
MR: Magnetic resonance
mRNA: Messenger ribonucleic acid
MWMOTE: Majority weighted minority over-sampling technique
NDCG: Normalized discounted cumulative gain
NIPS: Advances in neural information processing systems
NTL: Non-technical loss
ODT: Oblivious decision tree
OS: Optical sensor quasars quasi-stellar radio source
RF: Random forest
RFE: Recursive feature elimination
RGF: Regularized greedy forests
RMSE: Root mean squared error
RRF: Regularized random forest
RSME: Root mean squared error
RT: Regression tree
SDSS: Sloan digital sky survey
SHAP: Shapley additive explanations
SMO: Sequential minimal optimization
SMOTE: Synthetic minority over-sampling technique
SVC: Support vector classification
SVM: Support vector machine
TB: Terabytes
TF-IDF: Term frequency—inverse document frequency
TS: Target statistic

## Footnotes

1 https://www.csie.ntu.edu.tw/~cjlin/libsvmtools/datasets/binary.html.

2 https://catboost.ai/docs/concepts/python-reference_parameters-list.html#python-reference_parameters-list.

3 https://lendingclub.com

4 http://biometrics.idealtest.org/

5 http://www.itksnap.org
